# Supplementary material for: Circular single-stranded DNA as switchable vector for gene expression in mammalian cells
Source: Nat Commun. 2023 Oct 20;14:6665. doi: 10.1038/s41467-023-42437-6 (PMC10589306; doi:10.1038/s41467-023-42437-6)
Supplement: Supplementary file 1 — Supplementary Information [file 41467_2023_42437_MOESM1_ESM.pdf]

Supplementary Information for

Circular Single-stranded DNA as Switchable Vector for Gene Expression in Mammalian Cells

Linlin Tang†, Zhijin Tian†, Jin Cheng†, Yijing Zhang, Yongxiu Song, Yan Liu, Jinghao Wang, Pengfei Zhang, Yonggang Ke *, Friedrich C. Simmel *, and Jie Song *

†These authors contributed eqally.

*Email addresses for correspondence: sjie@sjtu.edu.cn; simmel@tum.de; yonggang.ke@emory.edu

This PDF file includes:

Supplementary Figures 1-37
Supplementary Tables 1-13

Supplementary Figures

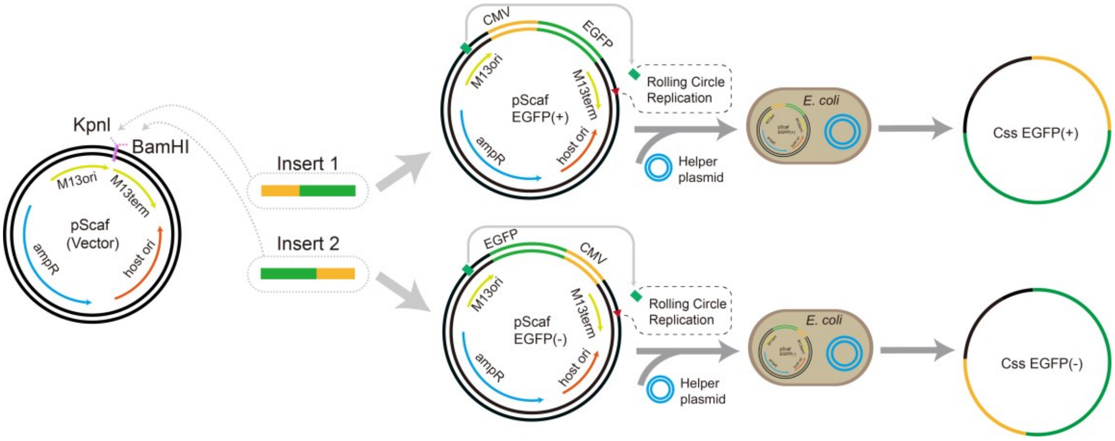

Supplementary Figure 1. Construction of the two different circular single-stranded DNAs used (Css EGFP(+), Css EGFP(-)) via the phagemid method. The pScaf phagemid contains a M13 origin sequence and a modified M13 origin sequence, which served for initiation of circular ssDNA synthesis and as terminator for the rolling circle replication, respectively. With the aid of helper-plasmid (pSB4423) co-transfected in XL1-Blue competent cell, the Css DNA phage can be produced in single-stranded form via intracellular rolling circle amplification in *E. coli* strains and then packaged and harvested in the culture supernatant, which can be purified by standard molecular biology operations for DNA purity. The schematic diagram was drawn with a reference of Figure 2 of the published paper by Shawn M. Douglas et al. (*Syn. Biol.* 2018, 3, ysy015).

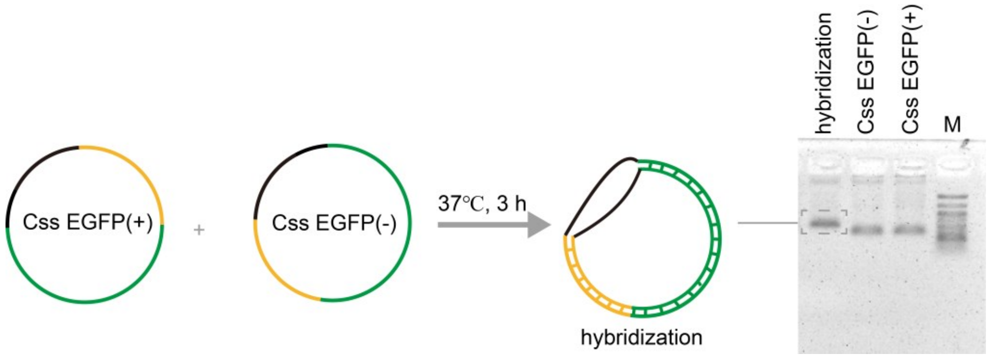

Supplementary Figure 2. Hybridization between Css EGFP(+) and Css EGFP(-) was conducted by incubating their mixtures with the same molar concentration (0.5 pmol, 50 μL) in TAE/Mg^{2+} buffer at 37°C for 3 h and the hybridization product was characterized by 1% agarose gel (run at 60 V for 1 h in TAE/Mg^{2+} buffer). For Css DNA, CMV promoter sequence and EGFP coding sequence are shown in yellow and green, respectively, and the black region is a custom sequence with a fixed region that is required for the production of the Css DNA via the pScaf phagemid method.

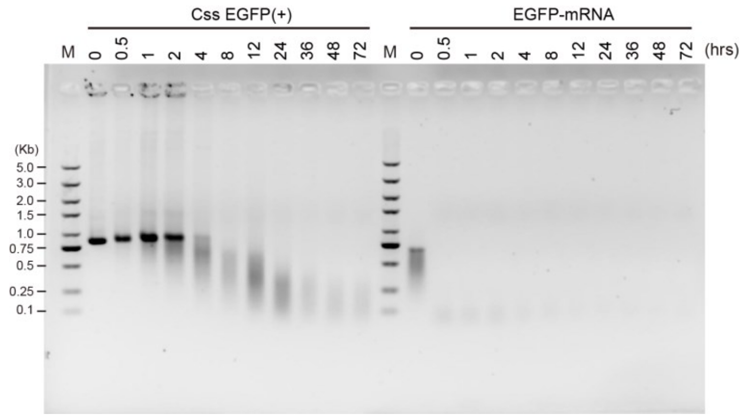

Supplementary Figure 3. Stability of Css EGFP(+) and EGFP-mRNA incubated in DMEM with 10% FBS at 37°C. We explored the biostability of Css DNA and EGFP-mRNA (a linear single-stranded mRNA for EGFP expression) in DMEM medium supplemented with 10% FBS. Css DNA and EGFP-mRNA were separately added to 20 μL of DMEM with 10% FBS (both at a weight of 400 ng), and the corresponding samples were incubated at 37 °C for a given time (0 h, 0.5 h, 1 h, 2 h, 4 h, 8 h, 12 h, 24 h, 36 h, 48 h and 72h, respectively). The analysis of all samples was conducted with a 1.0 % agarose gel, which was run for 1 h at a constant voltage of 80 V in TAE buffer. As shown in Supplementary Fig. 3, Css EGFP(+) was stable for up to 2 h, and then gradually degraded until it was completely degraded after 36 h. By contrast, EGFP-mRNA was completely degraded after 0.5 h, even the sample at 0 h without treatment in DMEM with 10% FBS would undergo a small amount of degradation. The results demonstrate that Css EGFP(+) is more stable than EGFP-mRNA in DMEM medium with 10% FBS at 37°C.

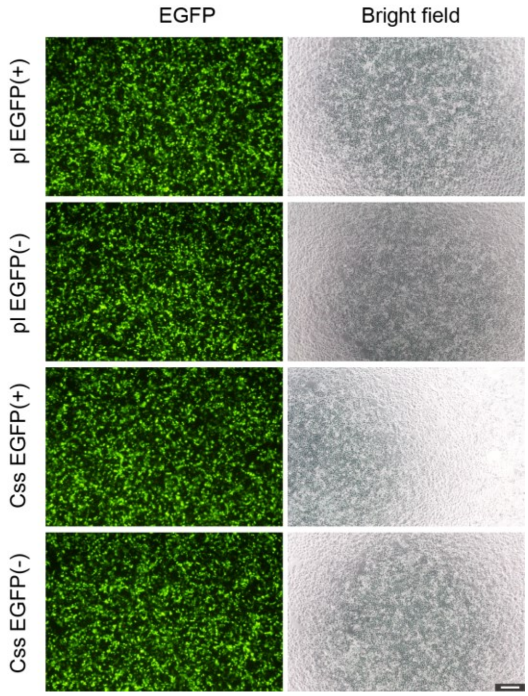

Supplementary Figure 4. Representative fluorescence microscopy images of cultured MDCK cells transfected with Css EGFP(+), Css EGFP(-), pl EGFP(+) and pl EGFP(-), respectively, after transfection for 24 h. The images are representative of one of n = 3 biologically independent experiments; similar results were observed each time. Scale bar, 100 μm.

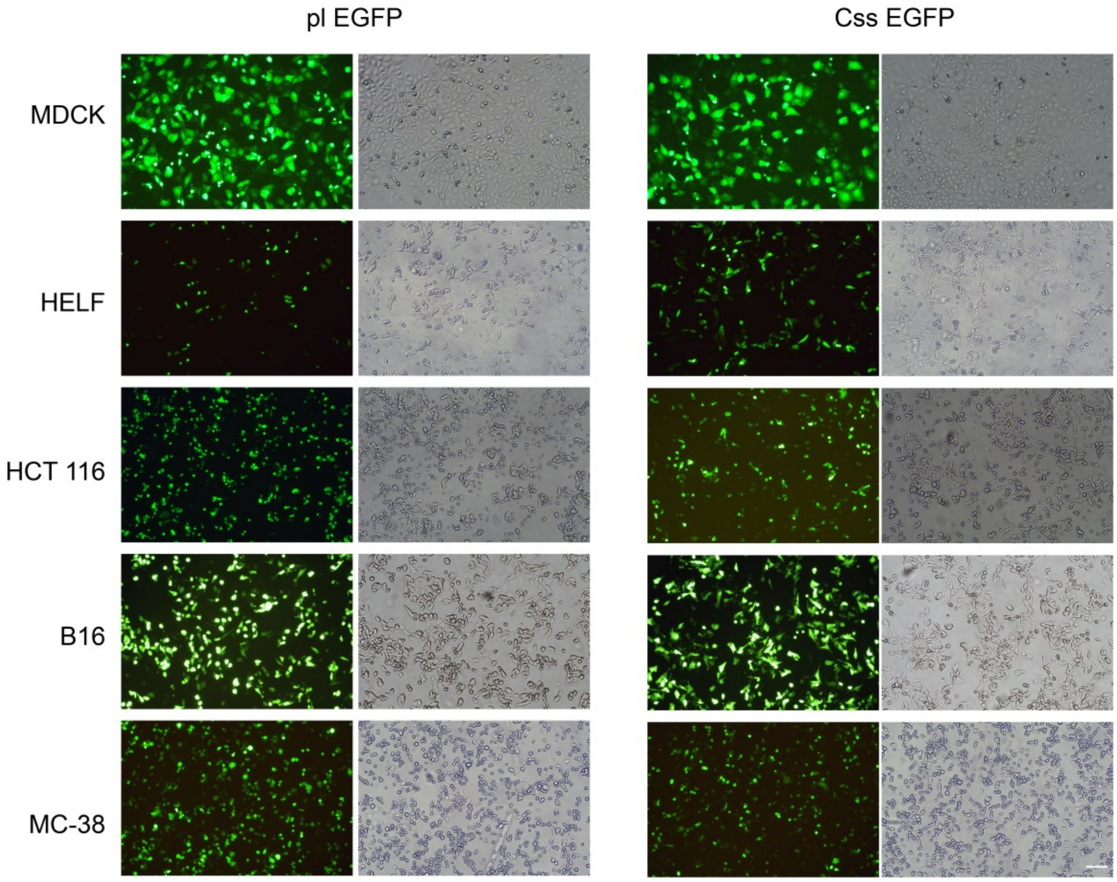

Supplementary Figure 5. Representative fluorescence microscopy images of various mammalian cells (MDCK, HELF, HCT 116, B16 and MC-38 cell lines, respectively) transfected with pl EGFP (0.5 pmol) and Css EGFP (0.5 pmol), respectively, by lip2000 (2 μL). The images are representative of one of n = 3 biologically independent experiments; similar results were observed each time. Scale bar, 100 μm.

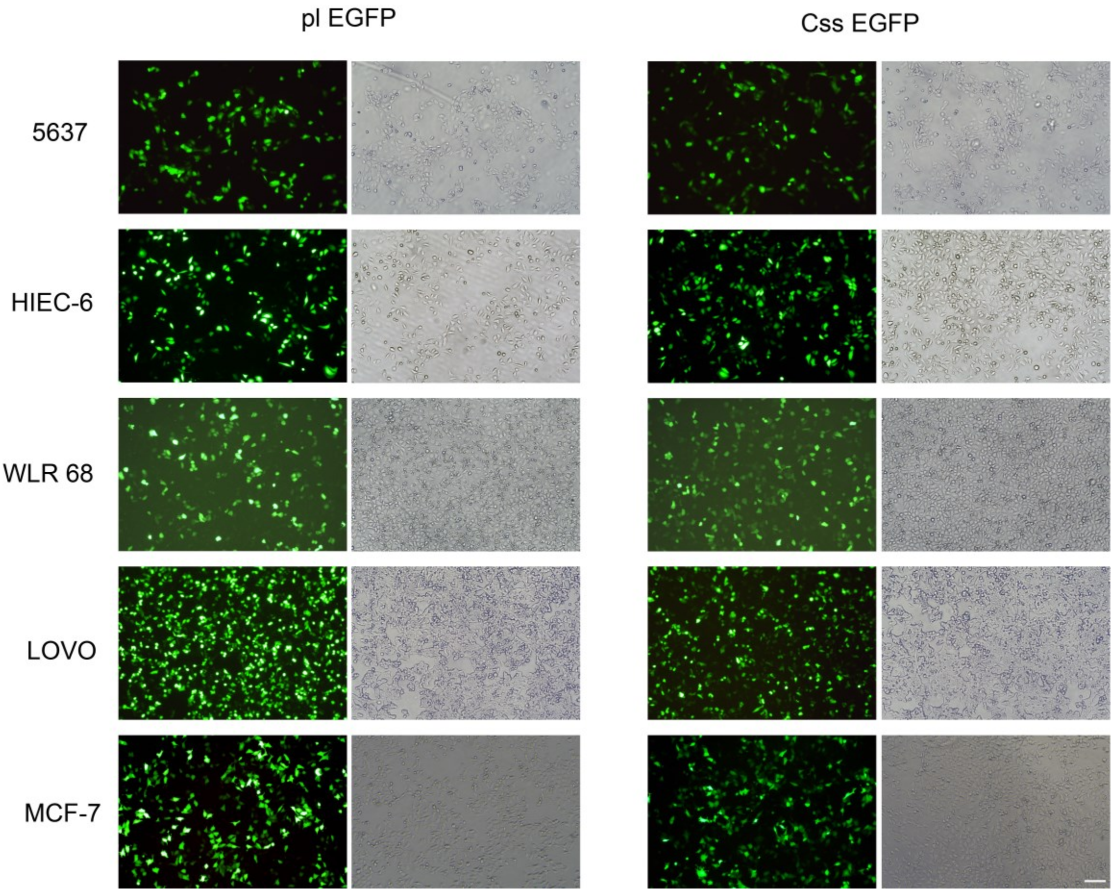

Supplementary Figure 6. Representative fluorescence microscopy images of various mammalian cells (5637, HIEC-6, WLR 68, LOVO and MCF-7 cell lines, respectively) transfected with pl EGFP (0.5 pmol) and Css EGFP (0.5 pmol), respectively, by lip2000 (2 μL). The images are representative of one of n = 3 biologically independent experiments; similar results were observed each time. Scale bar, 100 μm.

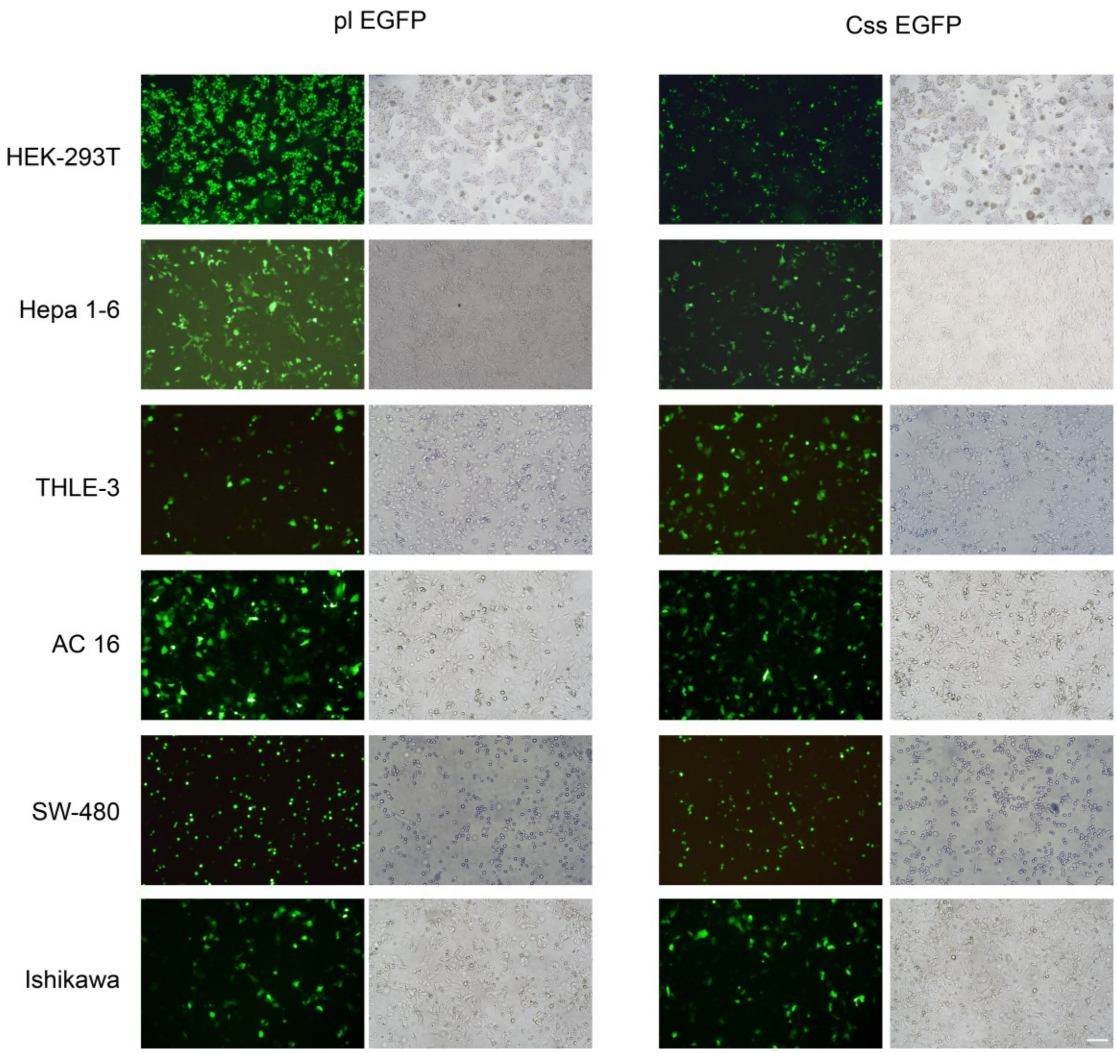

Supplementary Figure 7. Representative fluorescence microscopy images of various mammalian cells (HEK-293T, Hepa 1-6, THLE-3, AC 16, SW-480 and Ishikawa cell lines, respectively) transfected with pl EGFP (0.5 pmol) and Css EGFP (0.5 pmol), respectively, by lip2000 (2 μL). The images are representative of one of n = 3 biologically independent experiments; similar results were observed each time. Scale bar, 100 μm.

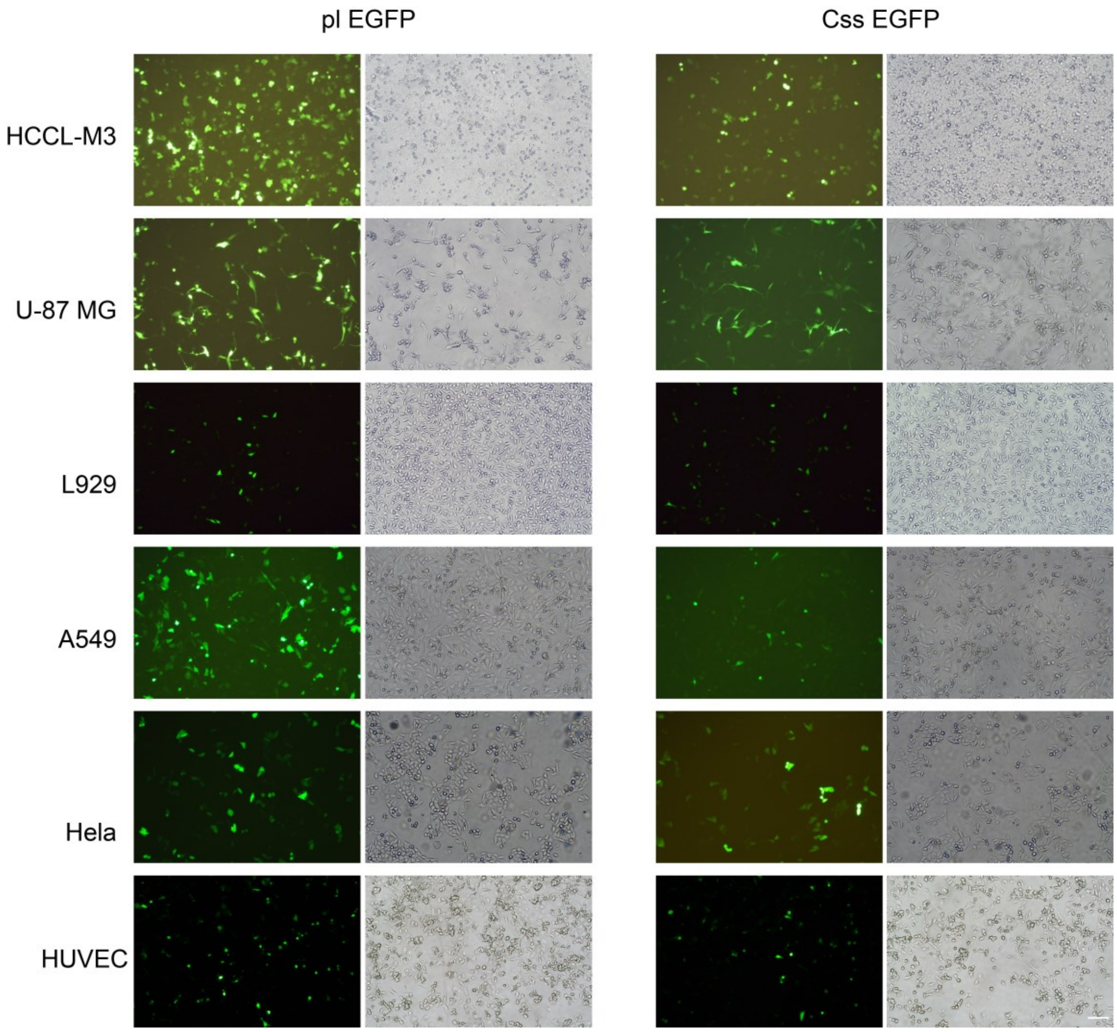

Supplementary Figure 8. Representative fluorescence microscopy images of various mammalian cells (HCCL-M3, U-87 MG, L929, A549, Hela and HUVEC cell lines, respectively) transfected with pl EGFP (0.5 pmol) and Css EGFP (0.5 pmol), respectively, by lip2000 (2 μL). The images are representative of one of n = 3 biologically independent experiments; similar results were observed each time. Scale bar, 100 μm.

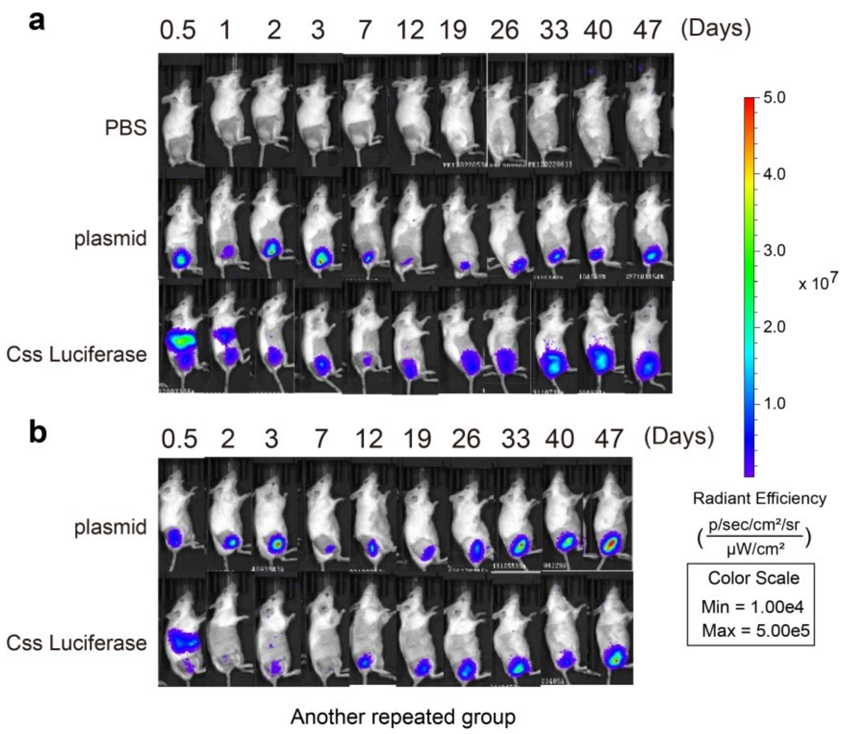

Supplementary Figure 9. In vivo luminescence images of mice after intramuscular administration of physiological saline (control), LNP-plasmid and LNP-Css Luciferase, respectively, for 0.5 - 47 days. **a** and **b** two independent tests for in vivo experiments. The LNPs were injected into the right thigh muscle. The Css Luciferase was mainly expressed in liver of mice, the plasmid Luciferase was mainly expressed at the injection site 12 h after injection, but both gene expression of Css DNA and plasmids then mainly occurred at the injection site from 2 – 47 days.

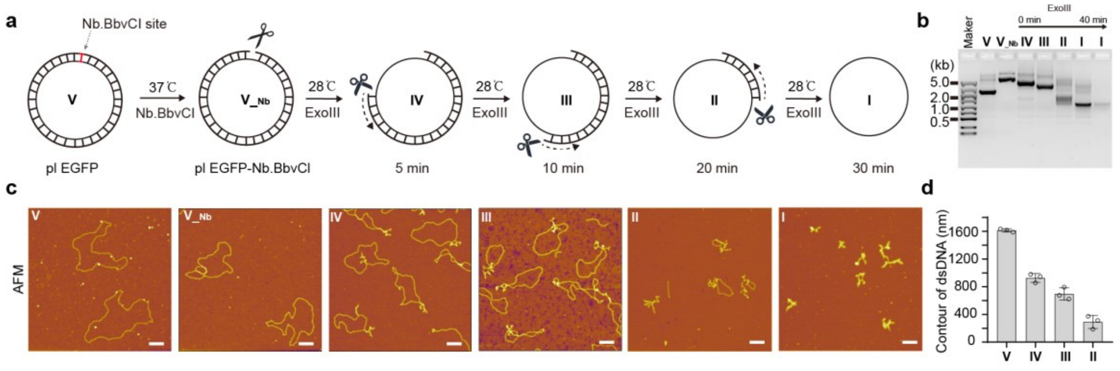

Supplementary Figure 10. Construction and characterization of Css DNA by Exonuclease III digestion. **a**. The process of the conversion of circular double-stranded DNA (pl DNA) to circular single-stranded DNA (Css DNA). The schematic diagram was drawn with a reference of Figure 1 of the published paper by Ibarra, B et al. (*Methods Mol. Biol.* 2021, 2281, 289-301). **b**. Agarose gel electrophoresis analysis (1%) of Css-ds DNA (V, V_{Nb}, IV, III, II and I). **c**. AFM images analysis of Css-ds DNA (V, V_{Nb}, IV, III, II and I). Scale bar, 100 nm. **d**. Statistical analysis of contour length of double-stranded DNA in Css-ds DNA (V, IV, III, and II) based on AFM images. The data are shown as the mean ± standard deviation (s.d.) of n = 3 independent experiments. Source data are provided as a Source Data file.

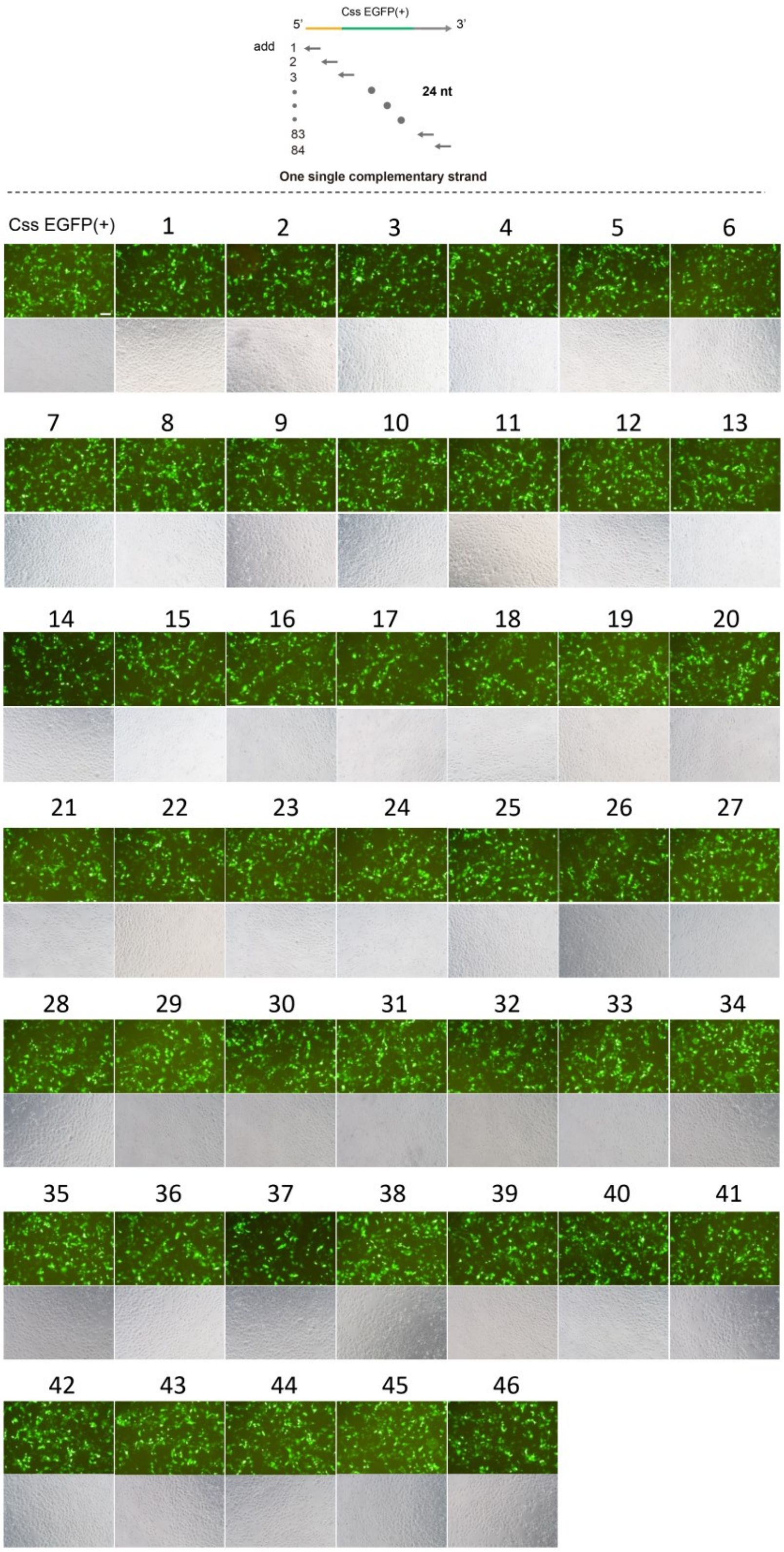

Supplementary Figure 11. The representative fluorescence images of cultured MDCK cells transfected with single Css EGFP(+) (0.5 pmol) or Css EGFP(+) hybridized by single complementary strands (Number 1-46, respectively, each 2.5 pmol) with the same length (24 nt). The images are representative of one of n = 3 biologically independent experiments; similar results were observed each time. Scale bar, 100 μm.

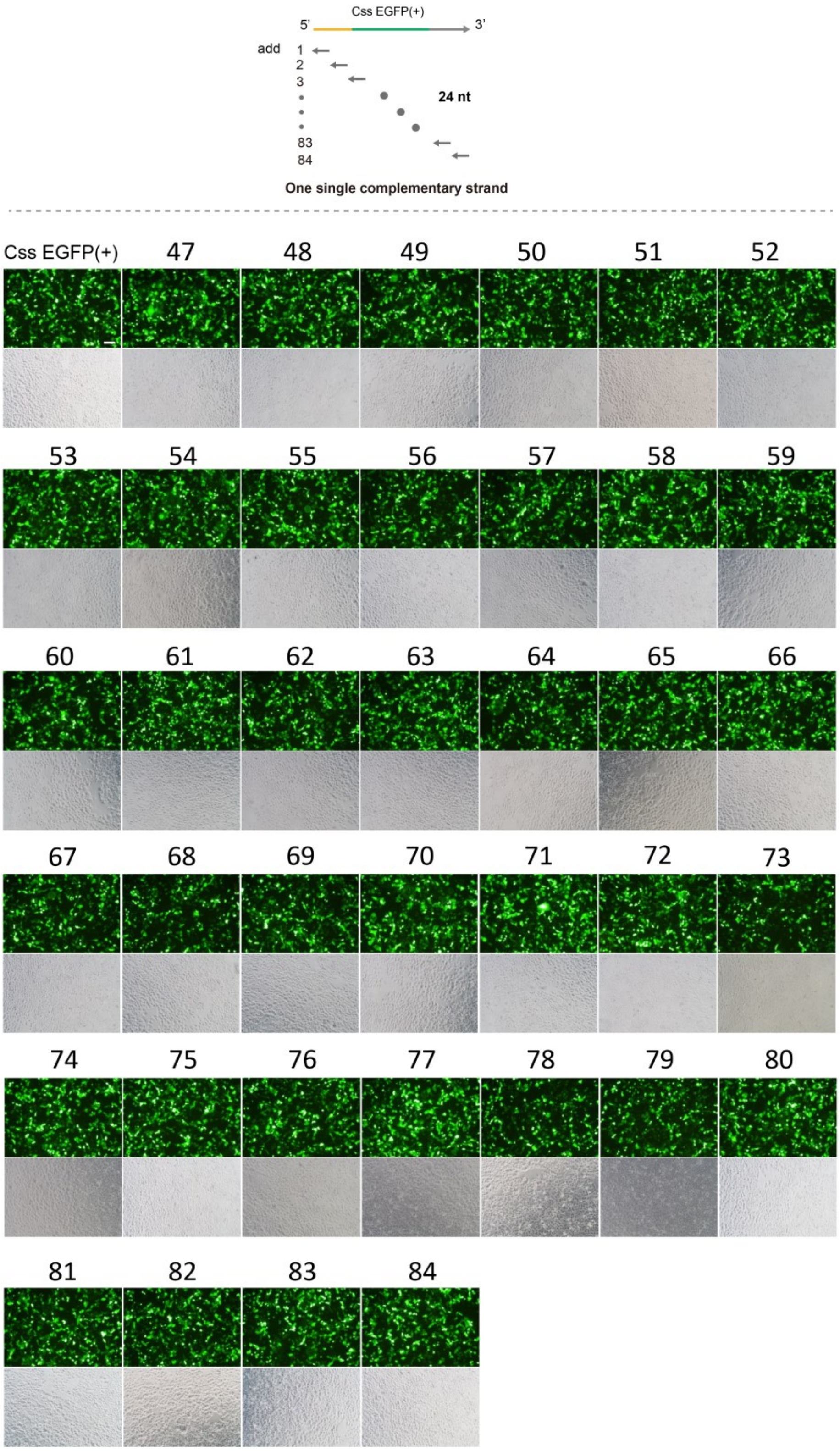

Supplementary Figure 12. The representative fluorescence images of cultured MDCK cells transfected with single Css EGFP(+) (0.5 pmol) or Css EGFP(+) hybridized by single complementary strands (Number 47-84, respectively, each 2.5 pmol) with the same length (24 nt). The images are representative of one of n = 3 biologically independent experiments; similar results were observed each time. Scale bar, 100 μm.

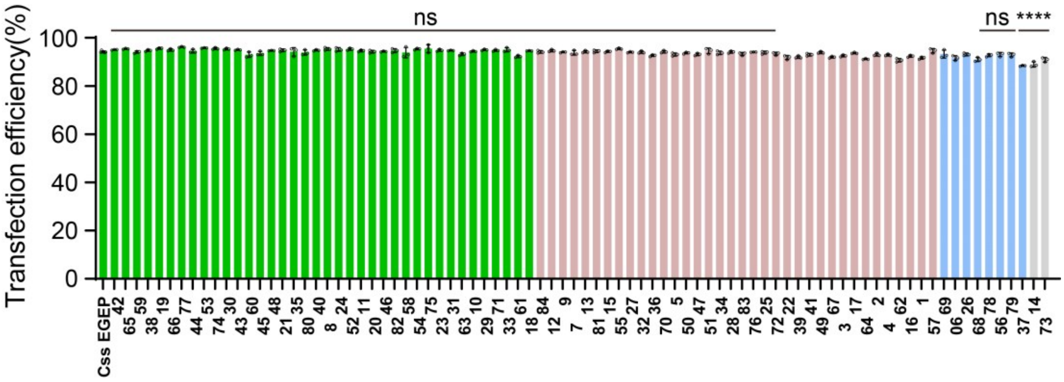

Supplementary Figure 13. Transfection efficiency of cultured MDCK cells transfected with single Css EGFP(+) (0.5 pmol) or Css EGFP(+) hybridized by single complementary strands (Number 1-84, respectively, each 2.5 pmol) with the same length (24 nt). Data collected were quantified using flow cytometry and are presented as mean ± standard deviation (s.d.) for n = 3 biologically independent experiments. Statistical analysis was performed using one-way ANOVA with Tukey's multiple comparison (****$p \leq 0.0001$, ns $p > 0.05$, ns indicates no significant difference). Source data are provided as a Source Data file. p-values are provided in the Source Data file.

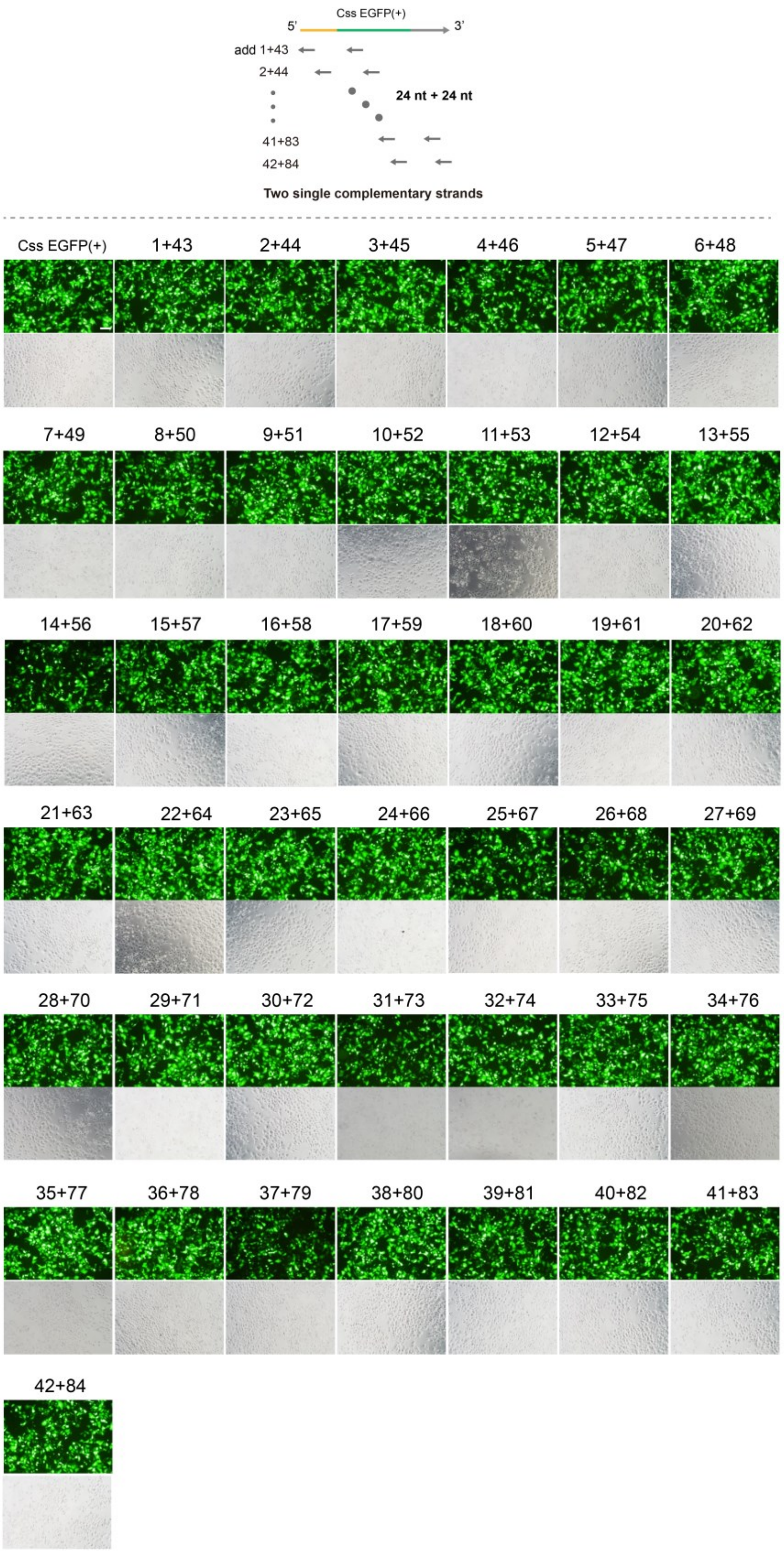

Supplementary Figure 14. The representative fluorescence images of cultured MDCK cells transfected with single Css EGFP(+) (0.5 pmol) or Css EGFP(+) hybridized by two single complementary strands (Number 1+43, 2+44, 3+45, …. , 42+84, respectively, each 2.5 pmol). The images are representative of one of n = 3 biologically independent experiments; similar results were observed each time. Scale bar, 100 μm.

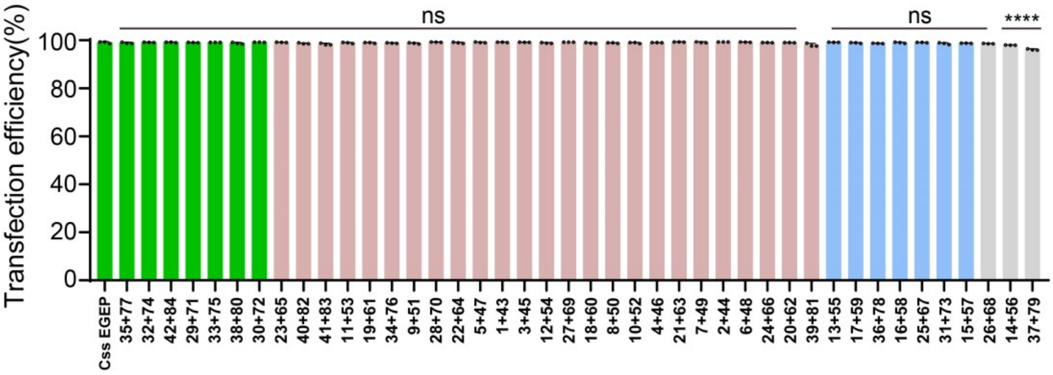

Supplementary Figure 15. Transfection efficiency of cultured MDCK cells transfected with single Css EGFP(+) (0.5 pmol) or Css EGFP(+) hybridized by two single complementary strands (Number 1+43, 2+44, 3+45, …. , 42+84, respectively, each 2.5 pmol). Data collected were quantified using flow cytometry and are presented as mean ± standard deviation (s.d.) for n = 3 biologically independent experiments. Statistical analysis was performed using one-way ANOVA with Tukey's multiple comparison (****$p \leq 0.0001$, ns $p > 0.05$, ns indicates no significant difference). Source data are provided as a Source Data file. p-values are provided in the Source Data file.

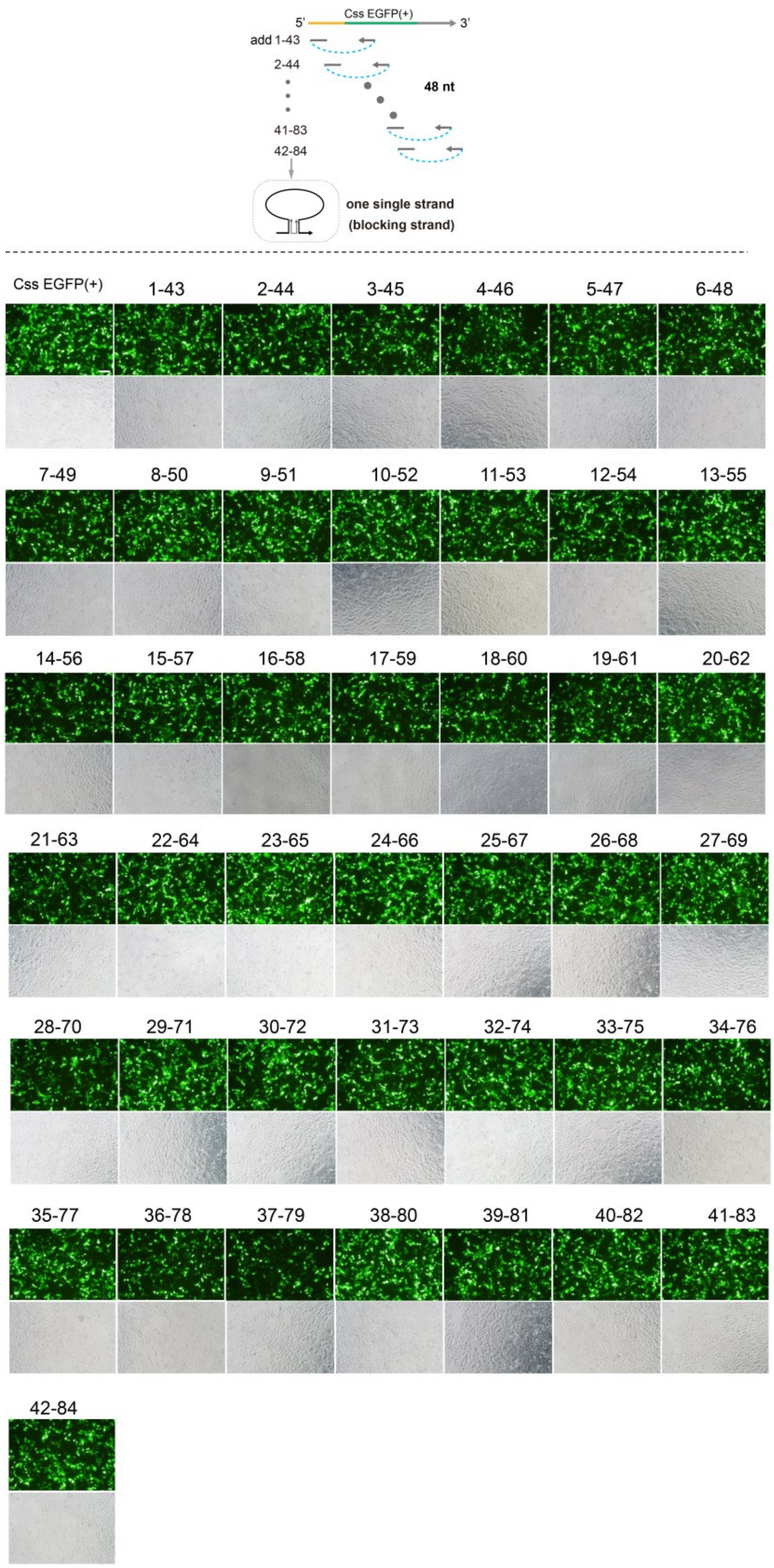

Supplementary Figure 16. The representative fluorescence images of cultured MDCK cells transfected with single Css EGFP(+) (0.5 pmol) or Css EGFP(+) hybridized by single fused strands (Number 1-43, 2-44, 3-45, …. , 42-84, respectively, each 2.5 pmol). The images are representative of one of n = 3 biologically independent experiments; similar results were observed each time. Scale bar, 100 μm.

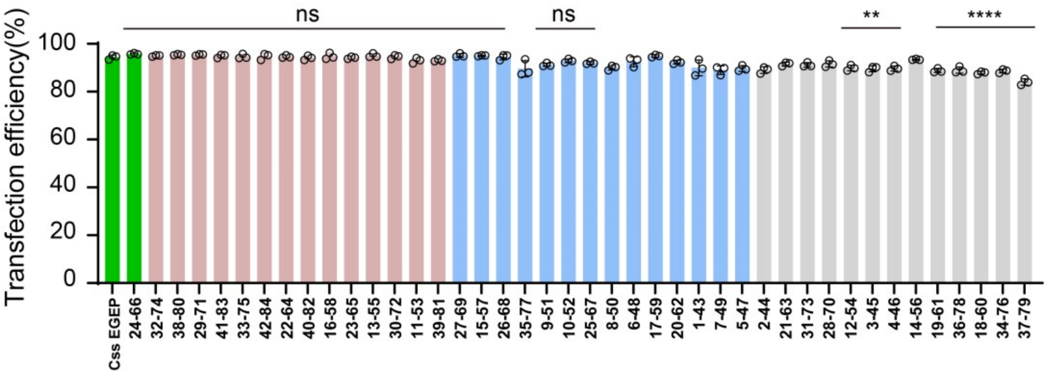

Supplementary Figure 17. Transfection efficiency of cultured MDCK cells transfected with single Css EGFP(+) (0.5 pmol) or Css EGFP(+) hybridized by single fused strands (Number 1-43, 2-44, 3-45, …. , 42-84, respectively, each 2.5 pmol). Data collected were quantified using flow cytometry and are presented as mean ± standard deviation (s.d.) for n = 3 biologically independent experiments. Statistical analysis was performed using one-way ANOVA with Tukey's multiple comparison (**$p \leq 0.01$, ****$p \leq 0.0001$, ns $p > 0.05$, ns indicates no significant difference). Source data are provided as a Source Data file. p-values are provided in the Source Data file.

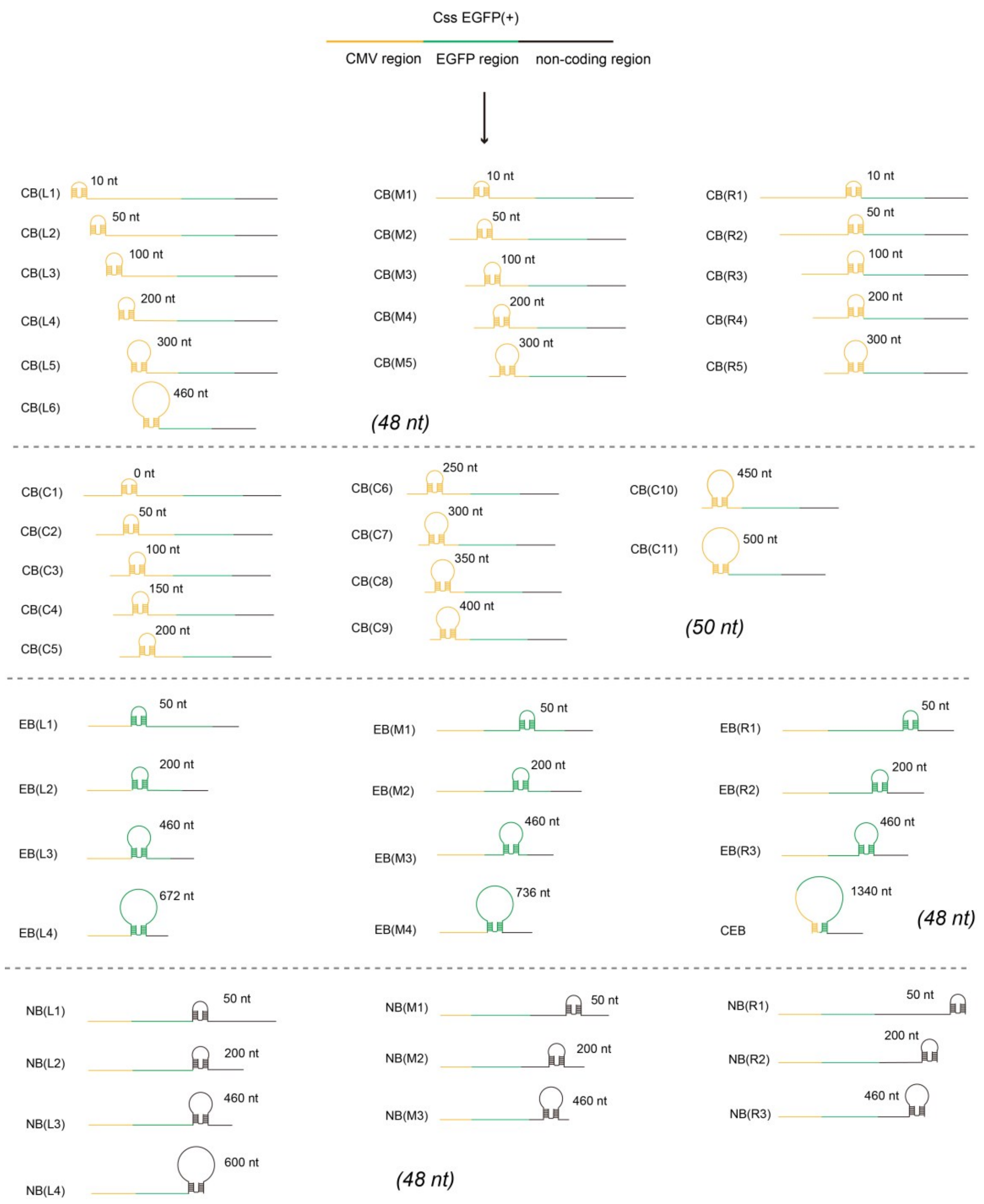

Supplementary Figure 18. Studying the influence of single blocking strands with the same length (48 nt or 50 nt) at different position of the CMV promoter (yellow), the EGFP coding region (green) and the non-coding region (black) on EGFP expression. We specifically targeted the CMV promoter, the EGFP coding region and the non-coding region of the Css DNA with blocking strands denoted CB, EB, and NB, respectively. For instance, CB(L1) is a 48 nt blocking strand that binds to 24 nt at the leftmost end of the CMV promoter and cross-links it with another 24 nt long domain in the promoter, creating a loop of 10 nt length in the Css DNA. In the same way, CB(L2), ... CB(L6) bind to the same leftmost domain, but connect them to sequences with increasing distance (and thus create larger loops up to 460 nt length). Correspondingly CB(M1) ... CB(M5) bind to the middle portion of the promoter, and CB(R1) ... CB(R5) bind to the rightmost part. Following the same scheme, crosslinker strands were also designed for the other regions, i.e., EB(L1) ... EB(L4),

EB(M1) ... EB(M4), EB(R1) ... EB(R3), NB(L1) ... NB(L4), NB(M1) ... NB(M3), NB(R1) ... NB(R3). In addition, CB(C1) … CB(C11) (50 nt) bind to the position near middle portion of the promoter. The single blocking strands (towards the different length of loop region, such as 10 nt, 50 nt, 100 nt, 200 nt, 300 nt and up to 1340 nt) were separately added to Css EGFP(+). Css EGFP(+) (0.5 pmol) and corresponding single-stranded blocking strand (2.5 pmol) were mixed in TE/10 mM Mg^{2+} (total 50 μL). These DNA samples were annealed 85°C for 5 min, from 85 to 37°C at the rate of 1°C/2 min, 37°C for 1 h, 12°C for 2 h.

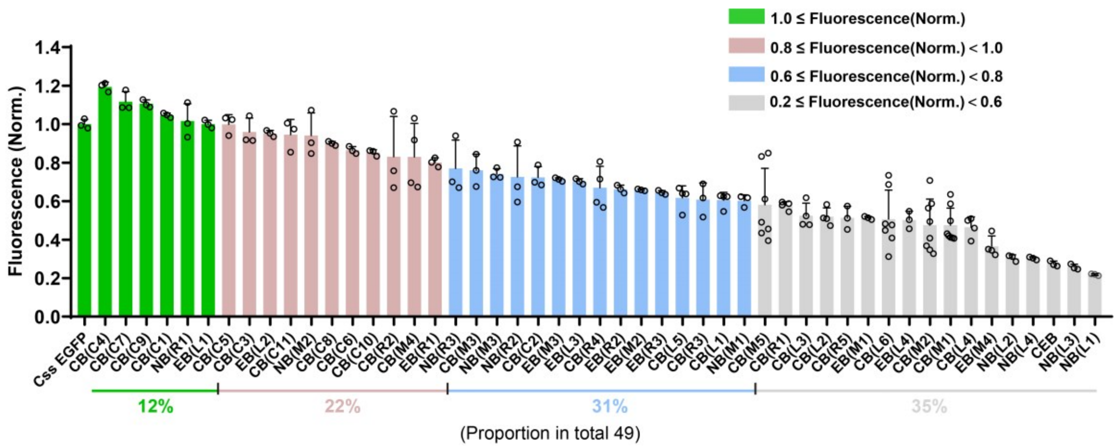

Supplementary Figure 19. Fluorescence intensity of cultured MDCK cells transfected with single Css EGFP(+) (0.5 pmol) or Css EGFP(+) hybridized by the corresponding blocking strands (a total of 49 strands) shown in Supplementary Figure 18 (each 2.5 pmol). Data collected were quantified using flow cytometry and are presented as mean ± standard deviation (s.d.) for n ≥ 3 biologically independent experiments. All fluorescence intensities were normalized to the value of the MDCK cell transfected with Css EGFP(+) (0.5 pmol). Source data are provided as a Source Data file.

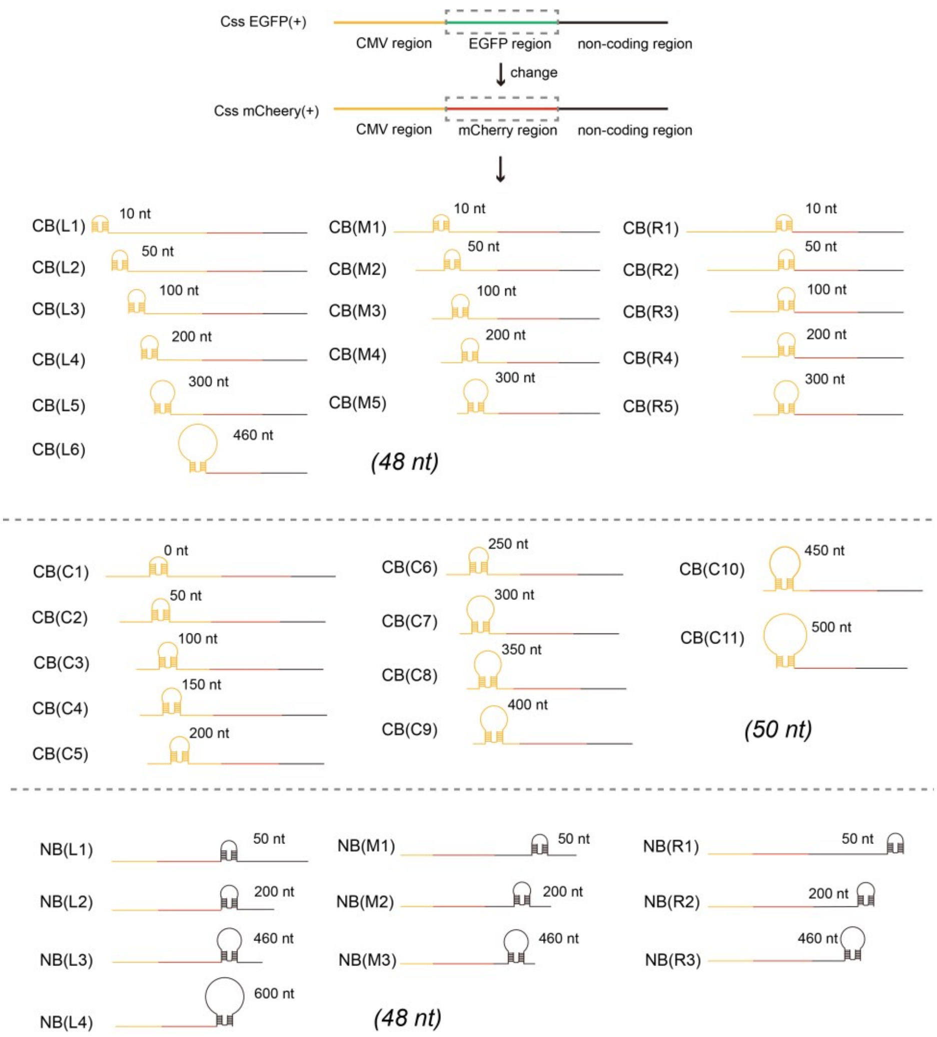

Supplementary Figure 20. Studying the influence of single blocking strands (48 nt or 50 nt) at different locations of the CMV promoter (yellow) and the non-coding region (black) on Css mCherry(+) expression. All sequences are consistent with those in Supplementary Figure 19. The single blocking strands (towards the different length of loop region, such as 10 nt, 50 nt, 100 nt, 200 nt, 300 nt and up to 600 nt) were separately added to Css mCherry(+). Css mCherry (+) (0.5 pmol) and corresponding single-stranded blocking strand (2.5 pmol) were mixed in TE/10 mM Mg^{2+} (total 50 μL). These DNA samples were annealed 85°C for 5 min, from 85 to 37°C at the rate of 1°C/2 min, 37°C for 1 h, 12°C for 2 h.

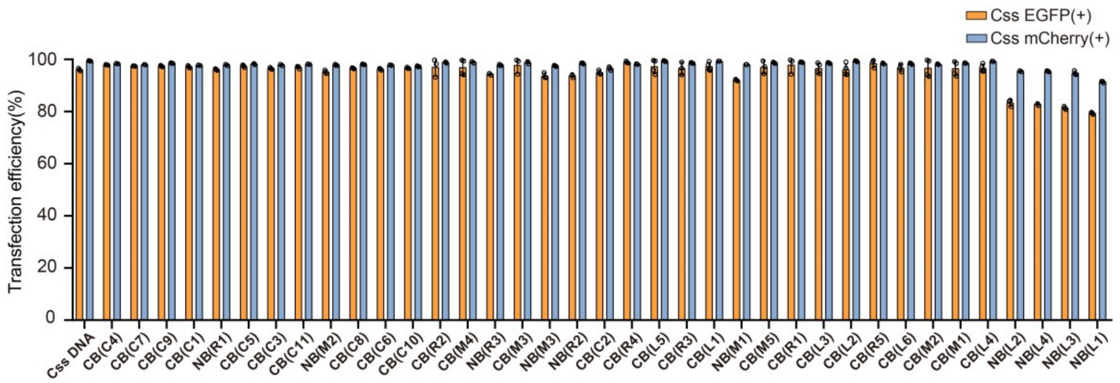

Supplementary Figure 21. Transfection efficiency of cultured MDCK cells transfected with single Css DNA (0.5 pmol) or Css DNA hybridized by the corresponding blocking strands shown in Supplementary Figure 20 (each 2.5 pmol). Data collected were quantified using flow cytometry and are presented as mean ± standard deviation (s.d.) for n = 3 biologically independent experiments. Source data are provided as a Source Data file.

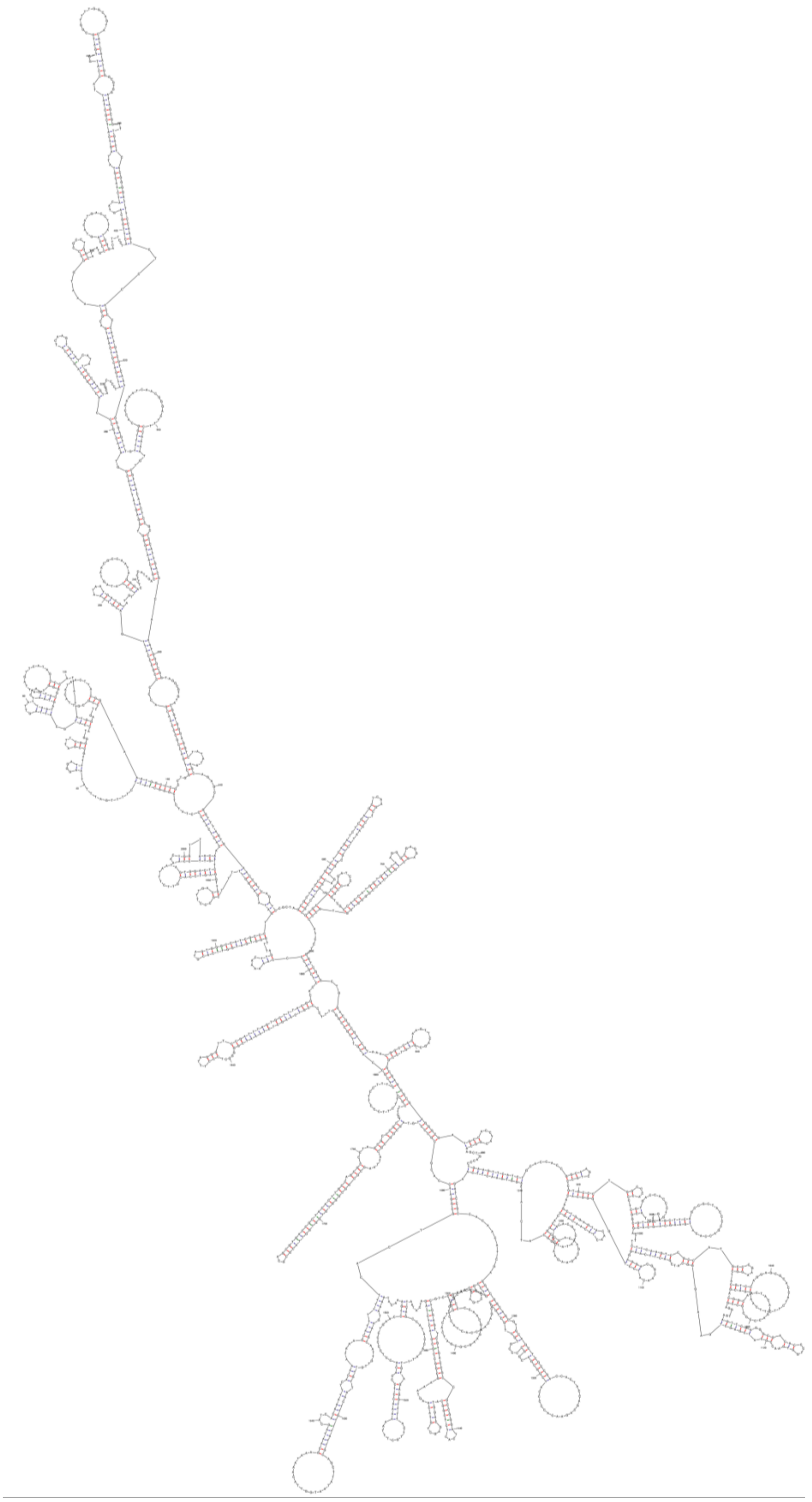

Supplementary Figure 22. The secondary structure prediction of Css EGFP(+) by UNAFold.

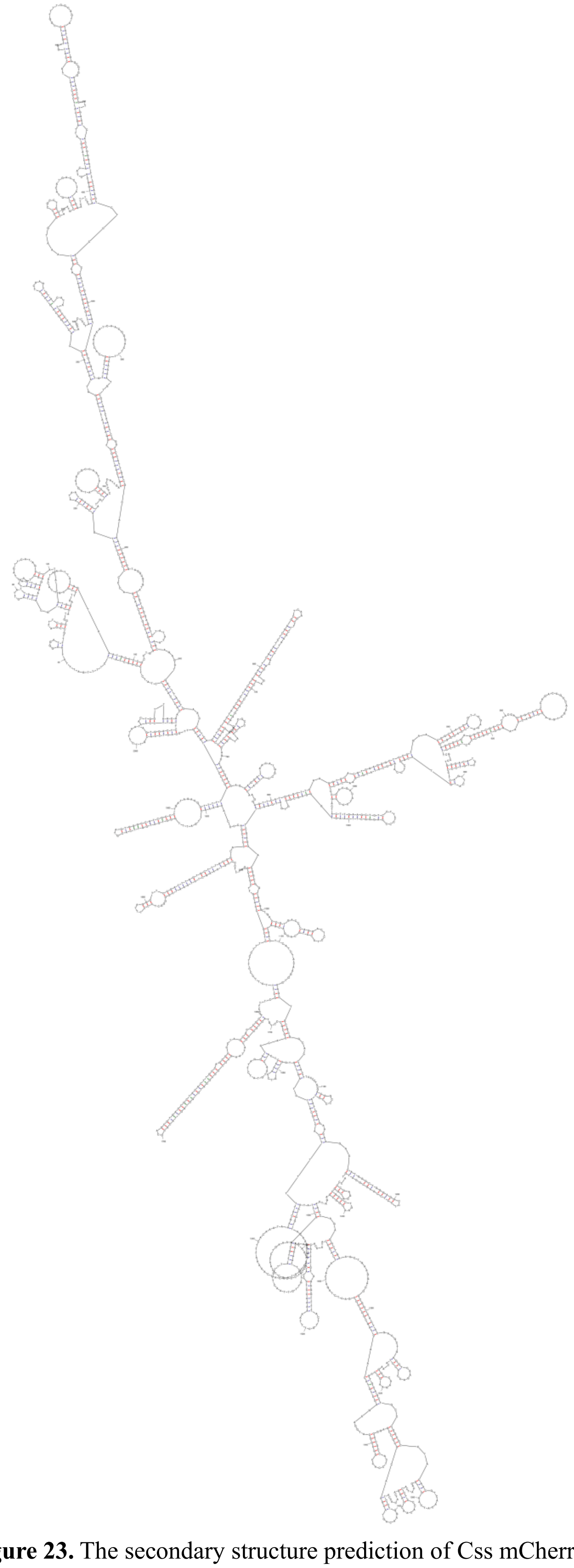

Supplementary Figure 23. The secondary structure prediction of Css mCherry(+) by UNAFold.

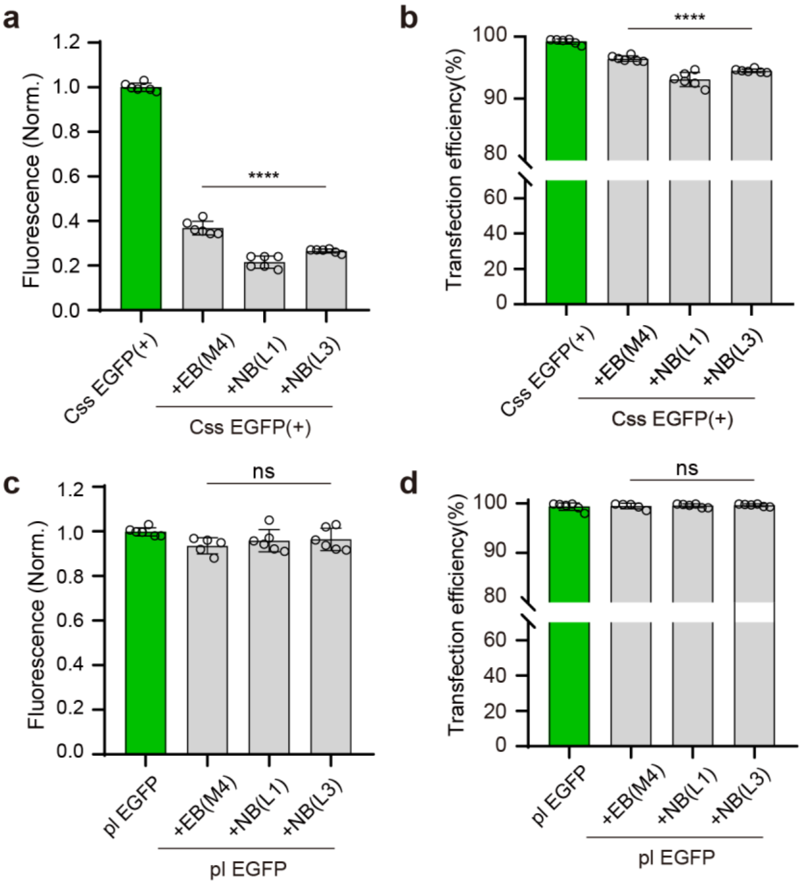

Supplementary Figure 24. Comparison of the inhibitory effects of the blocking strands on gene expression. **a** and **b**. Fluorescence intensity analysis and transfection efficiency, respectively, of cultured MDCK cells transfected with the corresponding products (Css EGFP(+) - blocking strand). **c** and **d**. fluorescence intensity analysis and transfection efficiency, respectively, of cultured MDCK cells transfected with the corresponding products (pl EGFP - blocking strand). All fluorescence intensities were normalized to the value of the corresponding mammalian cell transfected with Css EGFP(+) or pl EGFP alone. Data collected were quantified using flow cytometry and are presented as mean ± standard deviation (s.d.) for n = 6 biologically independent experiments, individual data points are overlaid. All fluorescence intensities were normalized to the fluorescence value of the corresponding mammalian cell transfected with the corresponding untreated pl EGFP or Css DNA. Statistical analysis was performed using one-way ANOVA with Tukey's multiple comparison (****$p \leq 0.0001$, ns $p > 0.05$, ns indicates no significant difference). Source data are provided as a Source Data file. p-values are provided in the Source Dada files.

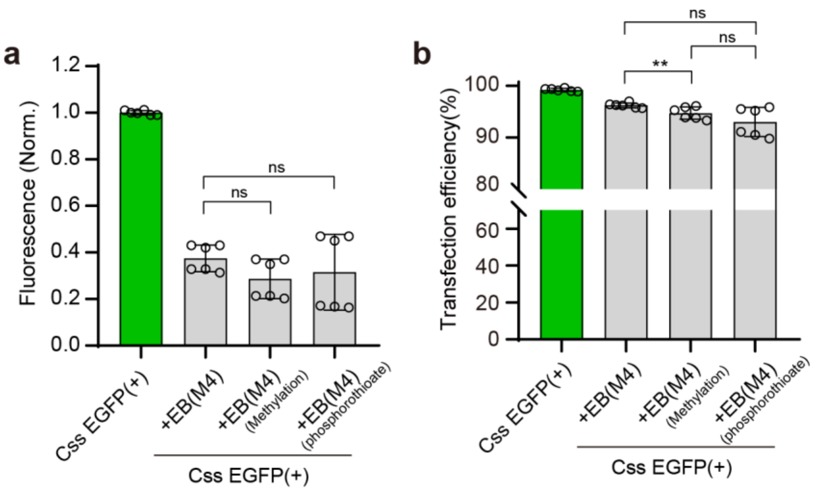

Supplementary Figure 25. Studying the influence of the chemical modification of 48 nt long single blocking strands of Css EGFP(+) on EGFP expression. **a** and **b**. Fluorescence intensity analysis and transfection efficiency, respectively, of cultured MDCK cells transfected with the corresponding products (Css EGFP(+) - blocking strand). Data collected were quantified using flow cytometry and are presented as mean ± standard deviation (s.d.) for n = 6 biologically independent experiments, individual data points are overlaid. Source data are provided as a Source Data file. All fluorescence intensities were normalized to the fluorescence value of the corresponding mammalian cell transfected with untreated Css EGFP(+). Statistical analysis was performed using one-way ANOVA with Tukey's multiple comparison (**$p \leq 0.01$, ns $p > 0.05$, ns indicates no significant difference). Source data are provided as a Source Data file. p-values are provided in the Source Dada files.

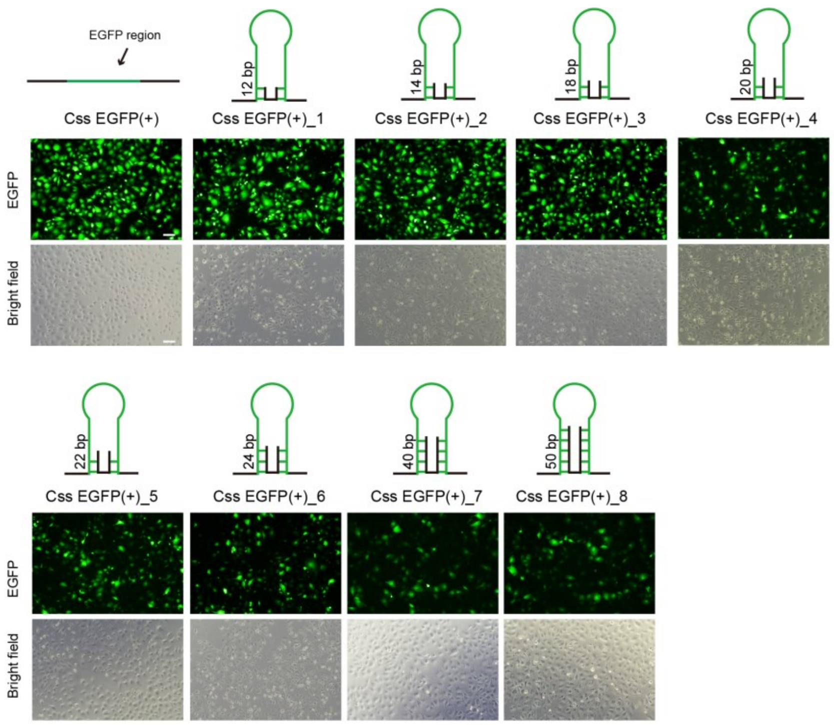

Supplementary Figure 26. Studying the influence of single blocking strands with the different length for Css EGFP(+) on EGFP expression. Css EGFP(+) (0.5 pmol) and single-stranded blocking strands (2.5 pmol) with different lengths (24 nt, 28 nt, 36 nt, 40 nt, 44 nt, 48 nt, 80 nt and 100 nt, respectively) were mixed in TE/10 mM Mg^{2+} (total 50 μL). These DNA samples were annealed 85°C for 5 min, from 85 to 37°C at the rate of 1°C/2 min, 37°C for 1 h, in which corresponding hybridization products (Css EGFP(+)_1/2/3/4/5/6/7/8) were transfected into cultured MDCK cells. The representative fluorescence images are recorded from MDCK cells transfected with untreated Css EGFP(+) or Css EGFP(+)_1/2/3/4/5/6/7/8, respectively. The images are representative of one of n = 3 biologically independent experiments; similar results were observed each time. Scale bar, 50 μm.

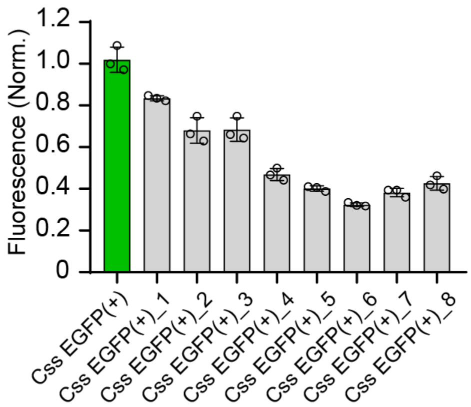

Supplementary Figure 27. The flow fluorescence analysis of cultured MDCK cells transfected (24 h) with Css EGFP(+) constructs with different blocking strands (Css EGFP(+)_1/2/3/4/5/6/7/8). Data collected were quantified using flow cytometry and are presented as mean ± standard deviation (s.d.) for n = 3 biologically independent experiments, individual data points are overlaid. All fluorescence intensities were normalized to the fluorescence value of the MDCK cells transfected with untreated Css EGFP(+). Source data are provided as a Source Data file.

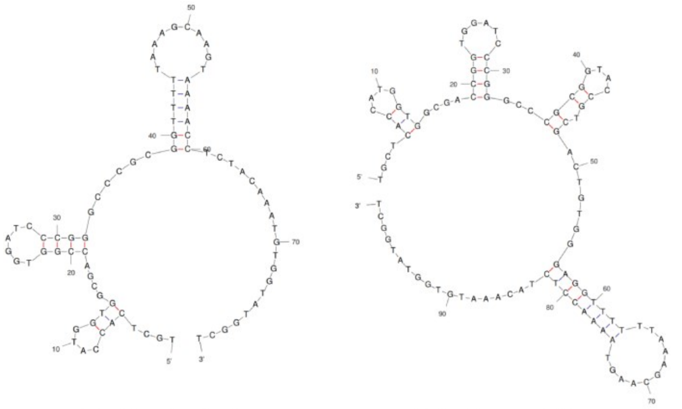

EGFP-block-80 nt　　EGFP-block-100 nt

Supplementary Figure 28. The secondary structure prediction of the longer blocking strands (EGFP-block-80 nt and EGFP-block-100 nt) by UNAFold.

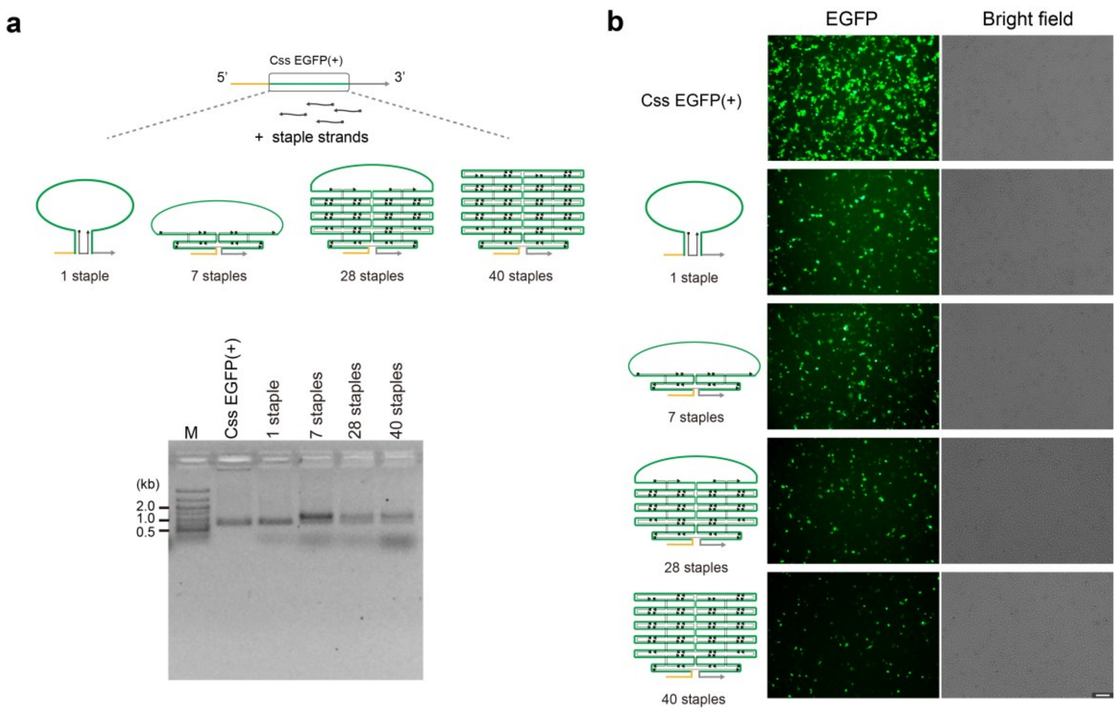

Supplementary Figure 29. The expression of Css EGFP(+) hybridized by a different number of staple strands**. a**. Different numbers of staples (1 staple, 7 staples, 28 staples and 40 staples) were added to the EGFP coding region of Css EGFP(+) to form the corresponding structures, which were characterized by a 1% agarose gel. **b**. The representative fluorescence images of cultured MDCK cells (24 h) transfected with single Css EGFP(+) (0.5 pmol), the blocked Css EGFP(+) (0.5 pmol) with 1 staple, 7 staples, 28 staples and 40 staples, respectively, via lipofection. The images are representative of one of n = 3 biologically independent experiments; similar results were observed each time. Scale bar, 100 µm.

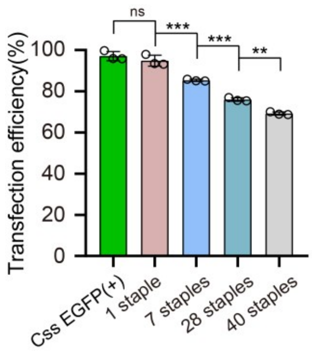

Supplementary Figure 30. The transfection efficiency analysis of cultured MDCK cells transfected with the corresponding products shown in Fig. 4e. Data collected were quantified using flow cytometry and are presented as mean ± standard deviation (s.d.) for n = 3 biologically independent experiments, individual data points are overlaid. Statistical analysis was performed using one-way ANOVA with Tukey's multiple comparison (**$p \leq 0.01$, ***$p \leq 0.001$, ns $p > 0.05$, ns indicates no significant difference). Source data are provided as a Source Data file. p-values are provided in the Source Dada files.

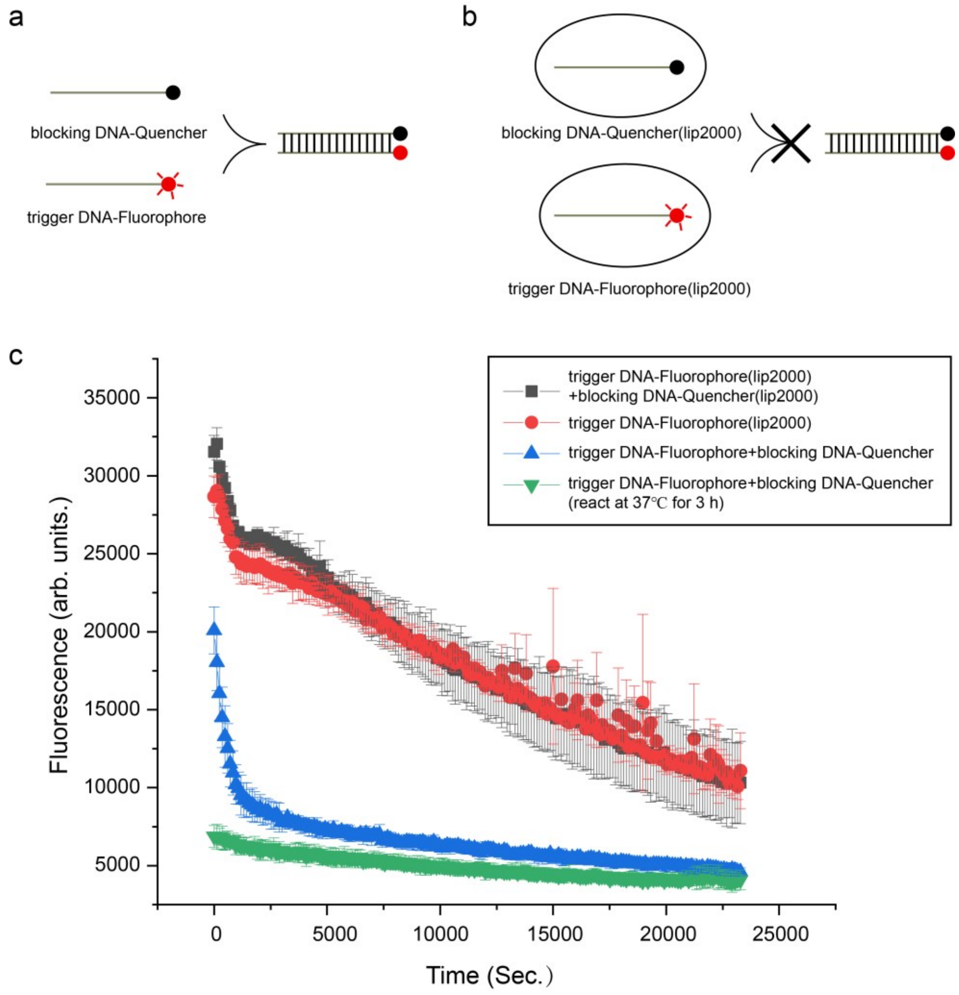

Supplementary Figure 31. In vitro mixing experiments. **a** and **b**. The scheme to illustrate that naked blocking DNA-Quencher and naked trigger DNA-Fluorophore can react by base pairing, but blocking DNA-Quencher and trigger DNA-Fluorophore were incubated separately with lip2000, in which the corresponding lipoplexes complex would not react with each other. **c**. The fluorescence intensity analysis about in vitro mixing experiments. The data were presented as mean ± standard deviation (s.d.) for n = 3 independent experiments.

First, the blocking DNA and trigger DNA were modified with Quencher (BHQ2) and Fluorophore (Cy3), respectively. When blocking DNA-Quencher and trigger DNA-Fluorophore react, the fluorescence intensity should decrease. Naked blocking DNA-Quencher and naked trigger DNA-Fluorophore (molar ratio, 1:1) were mixed, immediately detect the fluorescence intensity of the mixture (blue), the fluorescence intensity decreased significantly in 0-2000s, and after 2500s, which was consistent with that of the mixture of naked blocking DNA-Quencher and naked trigger DNA-Fluorophore were reacted at 37°C for 3 h (green), showing that the naked DNAs can be completely reacted within 2500s. In addition, the fluorescence intensity of lip2000-loaded trigger DNA-Fluorophore (red) would decrease slowly due to the self-quenching of Cy3. Similarly, the fluorescence intensity (black) from the complex of lip2000-loaded blocking DNA-Quencher and lip2000-loaded trigger DNA-Fluorophore was consistent with that of the trigger DNA-Fluorophore alone, indicating that that lipoplexes containing the blocked Css EGFP(+) and lipoplexes containing trigger DNA would not react with each other. (Sequences are given in Supplementary Table 13)

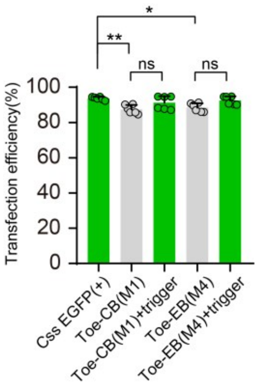

Supplementary Figure 32. Transfection efficiency of the circular single-stranded gene expression regulator (1-input YES). Data collected were quantified using flow cytometry and are presented as mean ± standard deviation (s.d.) for n = 3 biologically independent experiments, individual data points are overlaid. Statistical analysis was performed using one-way ANOVA with Tukey's multiple comparison (*$p \leq 0.05$, **$p \leq 0.01$, ns $p > 0.05$, ns indicates no significant difference). Source data are provided as a Source Data file. p-values are provided in the Source Dada files.

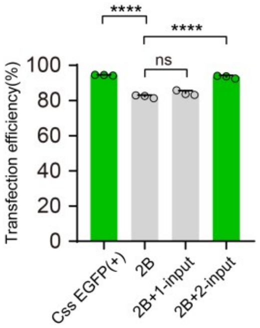

Supplementary Figure 33. Transfection efficiency of the circular single-stranded gene expression regulator based on Css EGFP(+) (2-input AND). Data collected were quantified using flow cytometry and are presented as mean ± standard deviation (s.d.) for n = 3 biologically independent experiments, individual data points are overlaid. Statistical analysis was performed using one-way ANOVA with Tukey's multiple comparison (****$p \leq 0.0001$, ns $p > 0.05$, ns indicates no significant difference). Source data are provided as a Source Data file. p-values are provided in the Source Dada files.

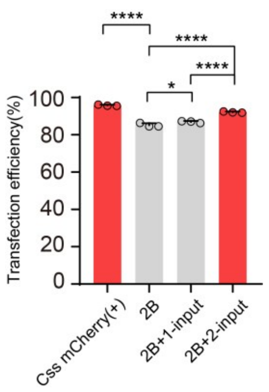

Supplementary Figure 34. Transfection efficiency of the circular single-stranded gene expression regulator based on Css mCherry(+) (2-input AND). Data collected were quantified using flow cytometry and are presented as mean ± standard deviation (s.d.) for n = 3 biologically independent experiments, individual data points are overlaid. Statistical analysis was performed using one-way ANOVA with Tukey's multiple comparison (*$p \leq 0.05$, ****$p \leq 0.0001$). Source data are provided as a Source Data file. p-values are provided in the Source Dada files.

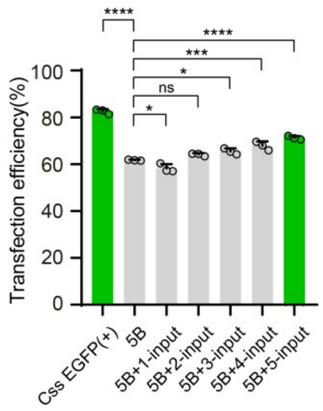

Supplementary Figure 35. Transfection efficiency of the circular single-stranded gene expression regulator based on Css EGFP(+) (5-input AND). Data collected were quantified using flow cytometry and are presented as mean ± standard deviation (s.d.) for n = 3 biologically independent experiments, individual data points are overlaid. Statistical analysis was performed using one-way ANOVA with Tukey's multiple comparison (*$p \leq 0.05$, ***$p \leq 0.001$, ****$p \leq 0.0001$, ns $p > 0.05$, ns indicates no significant difference). Source data are provided as a Source Data file. p-values are provided in the Source Dada files.

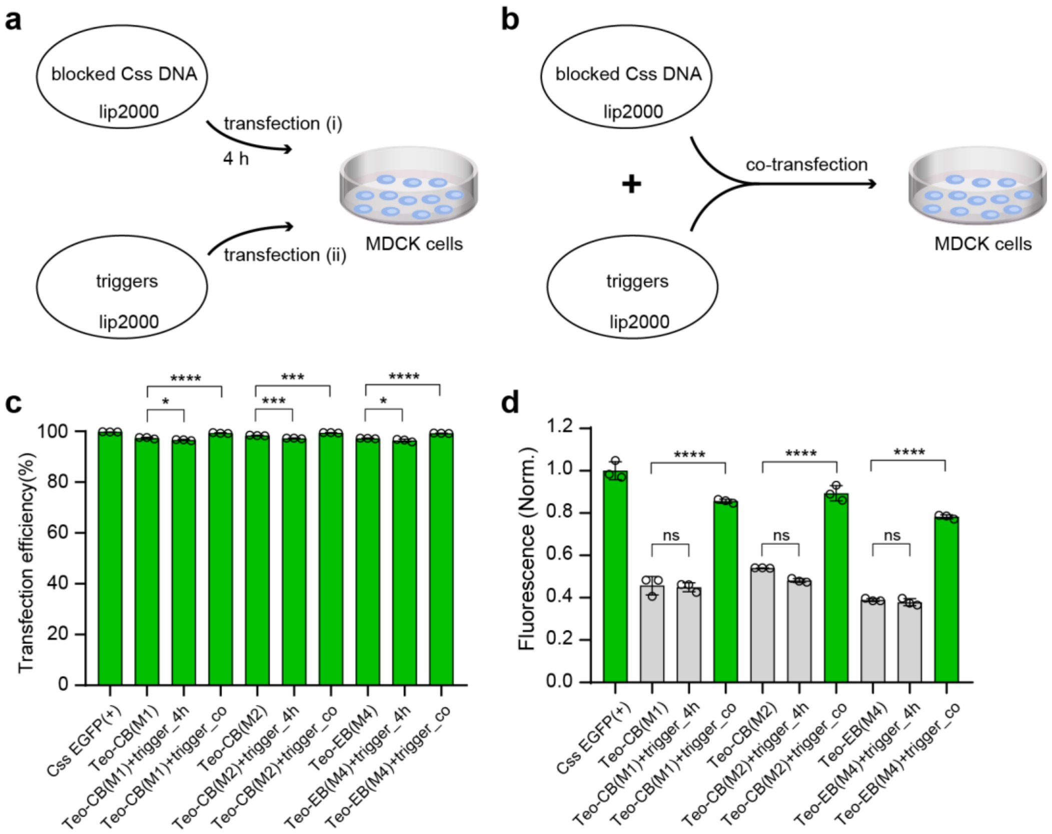

Supplementary Figure 36. Comparison of the regulatory effects of two different regulatory systems (**a** and **b**) on gene expression. Transfection efficiency (**c**) and fluorescence intensity analysis (**d**) of cultured MDCK cells through two different regulatory systems. Data collected were quantified using flow cytometry and are presented as mean ± standard deviation (s.d.) for n = 3 biologically independent experiments, individual data points are overlaid. Statistical analysis was performed using one-way ANOVA with Tukey's multiple comparison (*$p \leq 0.05$, ***$p \leq 0.001$, ****$p \leq 0.0001$, ns $p > 0.05$, ns indicates no significant difference). Source data are provided as a Source Data file. p-values are provided in the Source Dada files.

We used three "block-trigger" systems (CB(M1)_trigger; CB(M2)_trigger and EB(M4)_trigger, respectively) to explore the potential for a long-term dynamic regulation of gene expression. The lip2000-loaded blocked Css EGFP(+) construct was first transfected into MDCK cells, which was carried out at 37°C for 4 h, after which the transfection-medium was removed and replaced with the corresponding lip2000-loaded trigger strands (Supplementary Fig. 36a). The transfection results were monitored using flow cytometry after 24 h. As a control, the lip2000-loaded blocked Css EGFP(+) construct and lip2000-loaded trigger strands were co-transfected into MDCK cells (Supplementary Fig. 36b). As shown in Supplementary Fig. 36c, all transfections had a high transfection efficiency (above 95%). However, the fluorescence intensities (Supplementary Fig. 36d) indicate a difference in gene expression efficiency. The three blocked Css EGFP(+) constructs had 35% - 50% EGFP expression efficiency relative to the original Css EGFP(+) expression. When co-transfected with the corresponding trigger strand, they could obtain ~ 80% EGFP expression efficiency. By contrast, when the corresponding trigger strands are added later, after 4 h-transfection, there was no apparent change in the EGFP expression level.

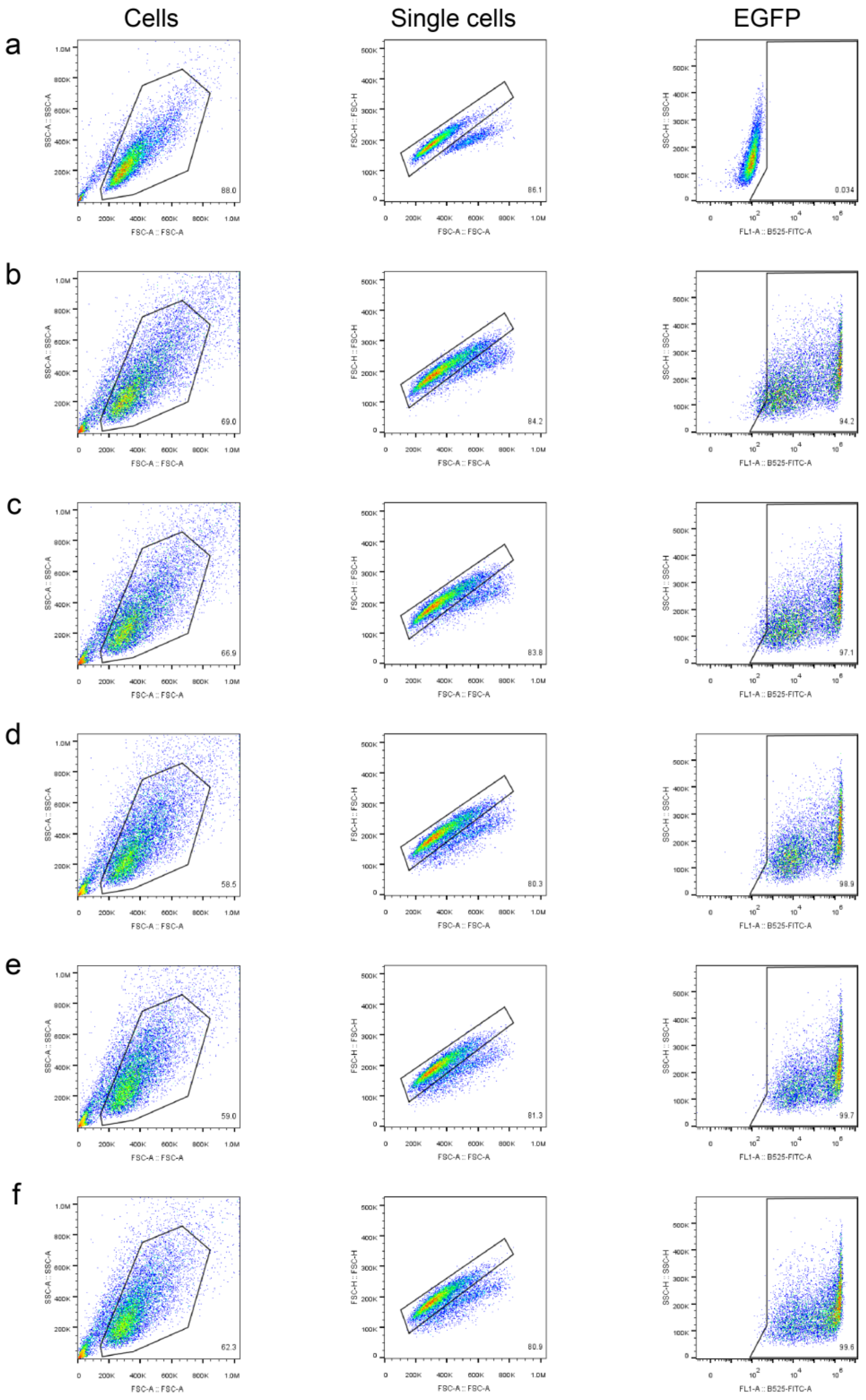

Supplementary Figure 37. Exemplary flow cytometry gating. From left to right: cell populations were first gated on forward scatter-area (FCS-A) versus side scatter-area (SSC-A), gate 'cells'; single cells were selected by gating FSC-A versus forward scatter-height (FSC-H), gate 'single cells'; cells were then assessed for EGFP expression. In this example, cell populations were investigated for transfection efficiency of EGFP expression (x-axis, EGFP fluorescence intensity). **a** gives representative gating for untreated cells, and transfection efficiency of samples **I-V** mentioned in Fig. 3b are given in **b-f**, respectively.

Supplementary Tables

Supplementary Table 1. Sequence of Css EGFP(+).

Css EGFP(+)
sequence (5’-3’)<br>aacaacactcaaccctatctcgggctattcttttgatttataagggattttgccgatttcggggtaccatgcattagttattaatagtaatcaattacgg<br>ggtcattagttcatagcccatatatggagttccgcgttacataacttacggtaaatggcccgcctggctgaccgcccaacgacccccgcccatt<br>gacgtcaataatgacgtatgttcccatagtaacgccaatagggactttccattgacgtcaatgggtggagtatttacggtaaactgcccacttgg<br>cagtacatcaagtgtatcatatgccaagtacgccccctattgacgtcaatgacggtaaatggcccgcctggcattatgcccagtacatgacctt<br>atgggactttcctacttggcagtacatctacgtattagtcatcgctattaccatggtgatgcggttttggcagtacatcaatgggcgtggatagcggt<br>ttgactcacggggatttccaagtctccaccccattgacgtcaatgggagtttgttttggcaccaaaatcaacgggactttccaaaatgtcgtaaca<br>actccgccccattgacgcaaatgggcggtaggcgtgtacggtgggaggtctatataagcagagctggtttagtgaaccgtcagatccgctag<br>cgctaccggactcagatctcgagctcaagcttcgaattctgcagtcgacggtaccgcgggcccgggatccaccggtcgccaccatggtgag<br>caagggcgaggagctgttcaccggggtggtgcccatcctggtcgagctggacggcgacgtaaacggccacaagttcagcgtgtccggcga<br>gggcgagggcgatgccacctacggcaagctgaccctgaagttcatctgcaccaccggcaagctgcccgtgccctggcccaccctcgtgac<br>caccctgacctacggcgtgcagtgcttcagccgctaccccgaccacatgaagcagcacgacttcttcaagtccgccatgcccgaaggctac<br>gtccaggagcgcaccatcttcttcaaggacgacggcaactacaagacccgcgccgaggtgaagttcgagggcgacaccctggtgaaccg<br>catcgagctgaagggcatcgacttcaaggaggacggcaacatcctggggcacaagctggagtacaactacaacagccacaacgtctatat<br>catggccgacaagcagaagaacggcatcaaggtgaacttcaagatccgccacaacatcgaggacggcagcgtgcagctcgccgaccac<br>taccagcagaacacccccatcggcgacggccccgtgctgctgcccgacaaccactacctgagcacccagtccgccctgagcaaagaccc<br>caacgagaagcgcgatcacatggtcctgctggagttcgtgaccgccgccgggatcactctcggcatggacgagctgtacaagtaaagcgg<br>ccgcgactctagatcataatcagccataccacatttgtagaggttttacttgctttaaaaaacctcccacacctccccctgaacctgaaacataa<br>aatgaatgcaattgttgttgttaacttgtttattgcagcttataatggttacaaataaagcaatagcatcacaaatttcacaaataaagcatttttttca<br>ctgcggatccacgcgccctgtagcggcgcattaagcgcggcgggtgtggtggttacgcgcagcgtgaccgctacacttgccagcgccctag<br>cgcccgctcctttcgctttcttcccttcctttctcgccacgttcgccggctttccccgtcaagctctaaatcgggggctccctttagggttccgatttagt<br>gctttacggcacctcgaccccaaaaaacttgatttgggtgatggttcacgtagtgggccatcgccctgatagacggtttttcgccctttgacgttgg<br>agtccacgttctttaatagtggactcttgttccaaactggtt

Supplementary Table 2. Sequence of Css EGFP(-).

Css EGFP(-)
Sequence (5’-3’)<br>gcagtgaaaaaaatgctttatttgtgaaatttgtgatgctattgctttatttgtaaccattataagctgcaataaacaagttaacaacaacaattgcat<br>tcattttatgtttcaggttcagggggaggtgtgggaggttttttaaagcaagtaaaacctctacaaatgtggtatggctgattatgatctagagtcgc<br>ggccgctttacttgtacagctcgtccatgccgagagtgatcccggcggcggtcacgaactccagcaggaccatgtgatcgcgcttctcgttggg<br>gtctttgctcagggcggactgggtgctcaggtagtggttgtcgggcagcagcacggggccgtcgccgatgggggtgttctgctggtagtggtcg<br>gcgagctgcacgctgccgtcctcgatgttgtggcggatcttgaagttcaccttgatgccgttcttctgcttgtcggccatgatatagacgttgtggct<br>gttgtagttgtactccagcttgtgccccaggatgttgccgtcctccttgaagtcgatgcccttcagctcgatgcggttcaccagggtgtcgccctcg<br>aacttcacctcggcgcgggtcttgtagttgccgtcgtccttgaagaagatggtgcgctcctggacgtagccttcgggcatggcggacttgaaga<br>agtcgtgctgcttcatgtggtcggggtagcggctgaagcactgcacgccgtaggtcagggtggtcacgagggtgggccagggcacgggca<br>gcttgccggtggtgcagatgaacttcagggtcagcttgccgtaggtggcatcgccctcgccctcgccggacacgctgaacttgtggccgtttac<br>gtcgccgtccagctcgaccaggatgggcaccaccccggtgaacagctcctcgcccttgctcaccatggtggcgaccggtggatcccgggcc<br>cgcggtaccgtcgactgcagaattcgaagcttgagctcgagatctgagtccggtagcgctagcggatctgacggttcactaaaccagctctgc<br>ttatatagacctcccaccgtacacgcctaccgcccatttgcgtcaatggggcggagttgttacgacattttggaaagtcccgttgattttggtgcca<br>aaacaaactcccattgacgtcaatggggtggagacttggaaatccccgtgagtcaaaccgctatccacgcccattgatgtactgccaaaacc<br>gcatcaccatggtaatagcgatgactaatacgtagatgtactgccaagtaggaaagtcccataaggtcatgtactgggcataatgccaggcg<br>ggccatttaccgtcattgacgtcaatagggggcgtacttggcatatgatacacttgatgtactgccaagtgggcagtttaccgtaaatactccacc<br>cattgacgtcaatggaaagtccctattggcgttactatgggaacatacgtcattattgacgtcaatgggcgggggtcgttgggcggtcagccag<br>gcgggccatttaccgtaagttatgtaacgcggaactccatatatgggctatgaactaatgaccccgtaattgattactattaataactaatgcatg<br>gggatccacgcgccctgtagcggcgcattaagcgcggcgggtgtggtggttacgcgcagcgtgaccgctacacttgccagcgccctagcgc<br>ccgctcctttcgctttcttcccttcctttctcgccacgttcgccggctttccccgtcaagctctaaatcgggggctccctttagggttccgatttagtgctt<br>tacggcacctcgaccccaaaaaacttgatttgggtgatggttcacgtagtgggccatcgccctgatagacggtttttcgccctttgacgttggagt<br>ccacgttctttaatagtggactcttgttccaaactggttaacaacactcaaccctatctcgggctattcttttgatttataaggattttgccgatttcgg<br>ggta

Supplementary Table 3. Sequence of Css mCherry(+).

Css mCherry(+)
sequence (5’-3’)<br>aacaacactcaaccctatctcgggctattcttttgatttataagggattttgccgatttcggggtaccatgcattagttattaatagtaatcaattacgg<br>ggtcattagttcatagcccatatatggagttccgcgttacataacttacggtaaatggcccgcctggctgaccgcccaacgacccccgcccatt<br>gacgtcaataatgacgtatgttcccatagtaacgccaatagggactttccattgacgtcaatgggtggagtatttacggtaaactgcccacttgg<br>cagtacatcaagtgtatcatatgccaagtacgccccctattgacgtcaatgacggtaaatggcccgcctggcattatgcccagtacatgacctt<br>atgggactttcctacttggcagtacatctacgtattagtcatcgctattaccatggtgatgcggttttggcagtacatcaatgggcgtggatagcggt<br>ttgactcacggggatttccaagtctccaccccattgacgtcaatgggagtttgttttggcaccaaaatcaacgggactttccaaaatgtcgtaaca<br>actccgccccattgacgcaaatgggcggtaggcgtgtacggtgggaggtctatataagcagagctggtttagtgaaccgtcagatccgctag<br>cgctaccggactcagatctcgagctcaagcttcgaattctgcagtcgacggtaccgcgggcccgggatccaccggtcgccaccatggtgag<br>caagggcgaggaggataacatggccatcatcaaggagttcatgcgcttcaaggtgcacatggagggctccgtgaacggccacgagttcga<br>gatcgagggcgagggcgagggccgcccctacgagggcacccagaccgccaagctgaaggtgaccaagggtggcccccctgcccttcgc<br>ctgggacatcctgtcccctcagttcatgtacggctccaaggcctacgtgaagcaccccgccgacatccccgactacttgaagctgtccttcccc<br>gagggcttcaagtgggagcgcgtgatgaacttcgaggacggcggcgtggtgaccgtgacccaggactcctccctgcaggacggcgagttc<br>atctacaaggtgaagctgcgcggcaccaacttcccctccgacggccccgtaatgcagaagaagaccatgggctgggaggcctcctccgag<br>cggatgtaccccgaggacggcgccctgaagggcgagatcaagcagaggctgaagctgaaggacggcggccactacgacgctgaggtc<br>aagaccacctacaaggccaagaagcccgtgcagctgcccggcgcctacaacgtcaacatcaagttggacatcacctcccacaacgagg<br>actacaccatcgtggaacagtacgaacgcgccgagggccgccactccaccggcggcatggacgagctgtacaagtagcggccgcgact<br>ctagatcataatcagccataccacatttgtagaggttttacttgctttaaaaaacctcccacacctccccctgaacctgaaacataaaatgaatg<br>caattgttgttgttaacttgtttattgcagcttataatggttacaaataaagcaatagcatcacaaatttcacaaataaagcatttttttcactgcattct<br>agttgtggtttgtccaaactcatcaatgtatcttaggatccacgcgccctgtagcggcgcattaagcgcggcgggtgtggtggttacgcgcagcg<br>tgaccgctacacttgccagcgccctagcgcccgctcctttcgctttcttcccttcctttctcgccacgttcgccggctttccccgtcaagctctaaatc<br>gggggctccctttagggttccgatttagtgctttacggcacctcgaccccaaaaaacttgatttgggtgatggttcacgtagtgggccatcgccct<br>gatagacggtttttcgccctttgacgttggagtccacgttctttaatagtggactcttgttccaaactggtt

Supplementary Table 4. Sequence of Css mCherry-EGFP(+).

Css mCherry-EGFP(+)
sequence (5’-3’)<br>aacaacactcaaccctatctcgggctattcttttgatttataagggattttgccgatttcggggtaccatgcattagttattaatagtaatcaattacgg<br>ggtcattagttcatagcccatatatggagttccgcgttacataacttacggtaaatggcccgcctggctgaccgcccaacgacccccgcccatt<br>gacgtcaataatgacgtatgttcccatagtaacgccaatagggactttccattgacgtcaatgggtggagtatttacggtaaactgcccacttgg<br>cagtacatcaagtgtatcatatgccaagtacgccccctattgacgtcaatgacggtaaatggcccgcctggcattatgcccagtacatgacctt<br>atgggactttcctacttggcagtacatctacgtattagtcatcgctattaccatggtgatgcggttttggcagtacatcaatgggcgtggatagcggt<br>ttgactcacggggatttccaagtctccaccccattgacgtcaatgggagtttgttttggcaccaaaatcaacgggactttccaaaatgtcgtaaca<br>actccgccccattgacgcaaatgggcggtaggcgtgtacggtgggaggtctatataagcagagctggtttagtgaaccgtcagatccgctag<br>cgctaccggactcagatctcgagccaccatggtgagcaagggcgaggaggataacatggccatcatcaaggagttcatgcgcttcaaggtg<br>cacatggagggctccgtgaacggccacgagttcgagatcgagggcgagggcgagggccgcccctacgagggcacccagaccgccaag<br>ctgaaggtgaccaagggtggccccctgcccttcgcctgggacatcctgtcccctcagttcatgtacggctccaaggcctacgtgaagcacccc<br>gccgacatccccgactacttgaagctgtccttccccgagggcttcaagtgggagcgcgtgatgaacttcgaggacggcggcgtggtgaccgt<br>gacccaggactcctccctgcaggacggcgagttcatctacaaggtgaagctgcgcggcaccaacttcccctccgacggccccgtaatgcag<br>aagaagaccatgggctgggaggcctcctccgagcggatgtaccccgaggacggcgccctgaagggcgagatcaagcagaggctgaag<br>ctgaaggacggcggccactacgacgctgaggtcaagaccacctacaaggccaagaagcccgtgcagctgcccggcgcctacaacgtca<br>acatcaagttggacatcacctcccacaacgaggactacaccatcgtggaacagtacgaacgcgccgagggccgccactccaccggcggc<br>atggacgagctgtacaagtagcggccgcgactctagatcataatcagccataccacatttgtagaggttttacttgctttaaaaaacctcccaca<br>cctccccctgaacctgaaacataaaatgaatgcaattgttgttgttaacttgtttattgcagcttataatggttacaaataaagcaatagcatcaca<br>aatttcacaaataaagcatttttttcactgcattctagttgtggtttgtccaaactcatcaatgtatcttaaggcgttagcccatatatggagttccgcgt<br>tacataacttacggtaaatggcccgcctggctgaccgcccaacgacccccgcccattgacgtcaataatgacgtatgttcccatagtaacgcc<br>aatagggactttccattgacgtcaatgggtggagtatttacggtaaactgcccacttggcagtacatcaagtgtatcatatgccaagtacgcccc<br>ctattgacgtcaatgacggtaaatggcccgcctggcattatgcccagtacatgaccttatgggactttcctacttggcagtacatctacgtattagt<br>catcgctattaccatggtgatgcggttttggcagtacatcaatgggcgtggatagcggtttgactcacggggatttccaagtctccaccccattga<br>cgtcaatgggagtttgttttggcaccaaaatcaacgggactttccaaaatgtcgtaacaactccgccccattgacgcaaatgggcggtaggcgt<br>gtacggtgggaggtctatataagcagagctggtttagtgaaccgtcagatccgctagcgccacctcgacggtaccgcgggcccgggatcca<br>ccggtcgccaccatggtgagcaagggcgaggagctgttcaccggggtggtgcccatcctggtcgagctggacggcgacgtaaacggcca<br>caagttcagcgtgtccggcgagggcgagggcgatgccacctacggcaagctgaccctgaagttcatctgcaccaccggcaagctgcccgt<br>gccctggcccaccctcgtgaccaccctgacctacggcgtgcagtgcttcagccgctaccccgaccacatgaagcagcacgacttcttcaagt<br>ccgccatgcccgaaggctacgtccaggagcgcaccatcttcttcaaggacgacggcaactacaagacccgcgccgaggtgaagttcgag<br>ggcgacaccctggtgaaccgcatcgagctgaagggcatcgacttcaaggaggacggcaacatcctggggcacaagctggagtacaacta<br>caacagccacaacgtctatatcatggccgacaagcagaagaacggcatcaaggtgaacttcaagatccgccacaacatcgaggacggc<br>agcgtgcagctcgccgaccactaccagcagaacacccccatcggcgacggccccgtgctgctgcccgacaaccactacctgagcaccca<br>gtccgccctgagcaaagaccccaacgagaagcgcgatcacatggtcctgctggagttcgtgaccgccgccgggatcactctcggcatgga<br>cgagctgtacaagtaaagcggccgcgactctagatcataatcagccataccacatttgtagaggttttacttgctttaaaaaacctcccacacct<br>cccccctgaacctgaaacataaaatgaatgcaattgttgttgttaacttgtttattgcagcttataatggttacaaataaagcaatagcatcacaaat<br>ttcacaaataaagcatttttttcactgcggatccacgcgccctgtagcggcgcattaagcgcggcgggtgtggtggttacgcgcagcgtgaccg<br>ctacacttgccagcgccctagcgcccgctcctttcgctttcttcccttcctttctcgccacgttcgccggctttccccgtcaagctctaaatcggggg<br>ctccctttagggttccgatttagtgctttacggcacctcgaccccaaaaaacttgatttgggtgatggttcacgtagtgggccatcgccctgataga<br>cggtttttcgccctttgacgttggagtccacgttctttaatagtggactcttgttccaaactggtt

Supplementary Table 5. Sequence of Css RFP-FUS(+).

Css RFP-FUS(+)
sequence (5’-3’)<br>aacaacactcaaccctatctcggctattcttttgatttataagggattttgccgatttcggggtaagggttattgtctcatgagcggatacatatttga<br>atgtatttagaaaaataaacaaataggggttccgcgcacatttccccgaaaagtgccacctgggtcgacattgattattgactagttattaatagt<br>aatcaattacggggtcattagttcatagcccatatatggagttccgcgttacataacttacggtaaatggcccgcctggctgaccgcccaacga<br>cccccgcccattgacgtcaataatgacgtatgttcccatagtaacgccaatagggactttccattgacgtcaatgggtggagtatttacggtaaa<br>ctgcccacttggcagtacatcaagtgtatcatatgccaagtacgccccctattgacgtcaatgacggtaaatggcccgcctggcattatgccca<br>gtacatgaccttatgggactttcctacttggcagtacatctacgtattagtcatcgctattaccatggtcgaggtgagccccacgttctgcttcactct<br>ccccatctcccccccctccccacccccaattttgtatttatttattttttaattattttgtgcagcgatgggggcggggggggggggggggcgcgcg<br>ccaggcggggcggggcggggcgaggggcggggcggggcgaggcgaaaaggtgcggcggcagccaatcagagcggcgcgctccga<br>aagtttccttttatggcgaggcggcggcggcggcggccctataaaaagcgaagcgcgcggcgggcgggagtcgctgcgcgctgccttcgc<br>cccgtgccccgctccgccgccgcctcgcgccgcccgccccggctctgactgaccgcgttactcccacaggtgagcgggcgggacggccctt<br>ctcctccgggctgtaattagcgcttggtttaatgacggcttgtttcttttctgtggctgcgtgaaagccttgaggggctccgggagggccctttgtgcg<br>ggggggagcggctcggggggtgcgtgcgtgtgtgtgtgcgtggggagcgccgcgtgcggctccgcgctgcccggcggctgtgagcgctgcg<br>ggcgcggcgcggggctttgtgcgctccgcagtgtgcgcgaggggagcgcggccgggggcggtgccccgcggtgcggggggggctgcga<br>ggggaacaaaggctgcgtgcggggtgtgtgcgtgggggggtgagcagggggtgtgggcgcgtcggtcgggctgcaaccccccctgcacc<br>cccctccccgagttgctgagcacggcccggcttcgggtgcggggctccgtacggggcgtggcgcggggctcgccgtgccgggcggggggt<br>ggcggcaggtgggggtgccgggcggggcggggccgcctcgggccggggagggctcgggggaggggcgcggcggcccccggagcgc<br>cggcggctgtcgaggcgcggcgagccgcagccattgccttttatggtaatcgtgcgagagggcgcagggacttcctttgtcccaaatctgtgc<br>ggagccgaaatctgggaggcgccgccgcaccccctctagcgggcgcggggcgaagcggtgcggcgccggcaggaaggaaatgggcg<br>gggagggccttcgtgcgtcgccgcgccgccgtccccttctccctctccagcctcggggctgtccgcgggggggacggctgccttcggggggga<br>cggggcagggcggggttcggcttctggcgtgtgaccggcggctctagagcctctgctaaccatgttcatgccttcttctttttcctacagctcctgg<br>gcaacgtgctggttattgtgctgtctcatcattttggcaaagaattccaccatggcctcctccgaggacgtcatcaaggagttcatgcgcttcaag<br>gtgcgcatggagggctccgtgaacggccacgagttcgagatcgagggcgagggcgagggccgcccctacgagggcacccagaccgcc<br>aagctgaaggtgaccaagggcggccccctgcccttcgcctgggacatcctgtcccctcagttccagtacggctccaaggcctacgtgaagca<br>ccccgccgacatccccgactacttgaagctgtccttccccgagggcttcaagtgggagcgcgtgatgaacttcgaggacggcggcgtggtga<br>ccgtgacccaggactcctccctgcaggacggcgagttcatctacaaggtgaagctgcgcggcaccaacttcccctccgacggccccgtaat<br>gcagaagaagaccatgggctgggaggcctccaccgagcggatgtaccccgaggacggcgccctgaagggcgagatcaagatgaggct<br>gaagctgaaggacggcggccactacgacgccgaggtcaagaccacctacatggccaagaagcccgtgcagctgcccggcgcctacaa<br>gaccgacatcaagctggacatcacctcccacaacgaggactacaccatcgtggaacagtacgagcgcgccgagggccgccactccacc<br>ggcgccggcggaagcatggcctcaaacgattatacccaacaagcaacccaaagctatggggcctaccccacccagcccgggcagggct<br>attcccagcagagcagtcagccctacggacagcagagttacagtggttatagccagtccacggacacttcaggctatggccagagcagcta<br>ttcttcttatggccagagccagaacacaggctatggaactcagtcaactccccagggatatggctcgactggcggctatggcagtagccaga<br>gctcccaatcgtcttacgggcagcagtcctcctaccctggctatggccagcagccagctcccagcagcacctcgggaagttacggtagcagtt<br>ctcagagcagcagctatgggcagccccagagtgggagctacagccagcagcctagctatggtggacagcagcaaagctatggacagca<br>gcaaagctataatccccctcagggctatggacagcagaaccagtacaacagcagcagtggtggtggaggtggaggtggaggtggaggta<br>actatggccaagatcaatcctccatgagtagtggtggtggcagtggtggcggttatggcaatcaagaccagagtggtggaggtggcagcggt<br>ggctatggacagcaggaccgtggaggccgcggcaggggtggcagtggtggcggcggcggcggcggtggtggttacaaccgcagc<br>agtggtggctatgaacccagaggtcgtggaggtggccgtggaggcagaggtggcatgggcggaagtgaccgtggtggcttcaataaatttg<br>gtggccctcgggaccaaggatcacgtcatgactccgaacaggataattcagacaacaacaccatctttgtgcaaggcctgggtgagaatgtt<br>acaattgagtctgtggctgattacttcaagcagattggtattattaagacaaacaagaaaacgggacagcccatgattaatttgtacacagaca<br>gggaaactggcaagctgaagggagaggcaacggtctcttttgatgacccaccttcagctaaagcagctattgactggtttgatggtaaagaat<br>tctccggaaatcctatcaaggtctcatttgctactcgccgggcagactttaatcggggtggtggcaatggtcgtggaggccgagggcgaggag<br>gacccatgggccgtggaggctatggaggtggtggcagtggtggtggtggccgaggaggatttcccagtggaggtggtggcggtggaggac<br>agcagcgagctggtgactggaagtgtcctaatcccacctgtgagaatatgaacttctcttggaggaatgaatgcaaccagtgtaaggcccctaa<br>aaccagatggcccaggagggggaccaggtggctctcacatgggggggtaactacggggatgatcgtcgtggtggcagaggaggctatgatc<br>gaggcggctaccggggccgcggcggggaccgtggaggcttccgagggggccggggtggtggggacagaggtggctttggccctggcaa<br>gatggattccaggggtgagcacagacaggatcgcagggagaggccgtattaatagctcgaggtacccccggggagctcaattcactcctca<br>ggtgcaggctgcctatcagaaggtggtggctggtgtggccaatgccctggctcacaaataccactgagatctttttccctctgccaaaaattatg<br>gggacatcatgaagccccttgagcatctgacttctggctaataaaggaaatttattttcattgcaatagtgtgttggaattttttgtgtctctcactcgg<br>aaggacatatgggagggcaaatcatggatccacgcgccctgtagcggcgcattaagcgcggcgggtgtggtggttacgcgcagcgtgacc<br>gctacacttgccagcgccctagcgcccgctcctttcgctttcttcccttcctttctcgccacgttcgccggctttccccgtcaagctctaaatcgggg<br>gctccctttagggttccgatttagtgctttacggcacctcgaccccaaaaaacttgatttgggtgatggttcacgtagtgggccatcgccctgatag<br>acggtttttcgccctttgacgttggagtccacgttctttaatagtggactcttgttccaaactggtt

Supplementary Table 6. Sequence of Css Luciferase(+).

Css Luciferase(+)
sequence (5'-3')<br>aacaacactcaaccctatctcgggctattcttttgatttataagggattttgccgatttcggggtaccatgcataataaaatatctttattttcattacat<br>ctgtgtgttggtttttgtgtgaatcgatagtactaacatacgctctccatcaaaacaaaacgaaacaaaacaaactagcaaaataggctgtccc<br>cagtgcaagtgcaggtgccagaacatttctctggcctaactggcctcaatattggccattagccatattattcattggttatatagcataaatcaat<br>attggctattggccattgcatacgttgtatctatatcataatatgtacatttatattggctcatgtccaatatgaccgccatgttggcattgattattgact<br>agttattaatagtaatcaattacggggtcattagttcatagcccatatatggagttccgcgttacataacttacggtaaatggcccgcctggctgac<br>cgcccaacgacccccgcccattgacgtcaataatgacgtatgttcccatagtaacgccaatagggactttccattgacgtcaatgggtggagt<br>atttacggtaaactgcccacttggcagtacatcaagtgtatcatatgccaagtccgccccctattgacgtcaatgacggtaaatggcccgcctg<br>gcattatgcccagtacatgaccttacgggactttcctacttggcagtacatctacgtattagtcatcgctattaccatggtgatgcggttttggcagta<br>caccaatgggcgtggatagcggtttgactcacggggatttccaagtctccaccccattgacgtcaatgggagtttgttttggcaccaaaatcaac<br>gggactttccaaaatgtcgtaataaccccgccccgttgacgcaaatgggcggtaggcgtgtacggtgggaggtctatataagcagagctcgtt<br>tagtgaaccgtcagatcactagaagctttattgcggtagtttatcacagttaaattgctaacgcagtcagtgggcctcggcggccaagcttggca<br>atccggtactgttggtaaagccaccatggaagatgccaaaaacattaagaagggcccagcgccattctacccactcgaagacgggaccgc<br>cggcgagcagctgcacaaagccatgaagcgctacgccctggtgcccggcaccatcgcctttaccgacgcacatatcgaggtggacattac<br>ctacgccgagtacttcgagatgagcgttcggctggcagaagctatgaagcgctatgggctgaatacaaaccatcggatcgtggtgtgcagcg<br>agaatagcttgcagttcttcatgcccgtgttgggtgccctgttcatcggtgtggctgtggccccagctaacgacatctacaacgagcgcgagctg<br>ctgaacagcatgggcatcagccagcccaccgtcgtattcgtgagcaagaaagggctgcaaaagatcctcaacgtgcaaaagaagctacc<br>gatcatacaaaagatcatcatcatggatagcaagaccgactaccagggcttccaaagcatgtacaccttcgtgacttcccatttgccacccgg<br>cttcaacgagtacgacttcgtgcccgagagcttcgaccgggacaaaaccatcgccctgatcatgaacagtagtggcagtaccggattgccca<br>agggcgtagccctaccgcaccgcaccgcttgtgtccgattcagtcatgcccgcgaccccatcttcggcaaccagatcatccccgacaccgct<br>atcctcagcgtggtgccatttcaccacggcttcggcatgttcaccacgctgggctacttgatctgcggctttcgggtcgtgctcatgtaccgcttcg<br>aggaggagctattcttgcgcagcttgcaagactataagattcaatctgccctgctggtgcccacactatttagcttcttcgctaagagcactctcat<br>cgacaagtacgacctaagcaacttgcacgagatcgccagcggcggggcgccgctcagcaaggaggtaggtgaggccgtggccaaacg<br>cttccacctaccaggcatccgccagggctacggcctgacagaaacaaccagcgccattctgatcacccccgaaggggacgacaagcctg<br>gcgcagtaggcaaggtggtgcccttcttcgaggctaaggtggtggacttggacaccggtaagacactgggtgtgaaccagcgcggcgagct<br>gtgcgtccgtggccccatgatcatgagcggctacgttaacaaccccgaggctacaaacgctctcatcgacaaggacggctggctgcacagc<br>ggcgacatcgcctactgggacgaggacgagcacttcttcatcgtggaccggctgaagagcctgatcaaatacaagggctaccaggtagcc<br>ccagccgaactggagagcatcctgctgcaacaccccaacatcttcgacgccggggtcgccggcctgcccgacgacgatgccggcgagct<br>gcccgccgcagtcgtcgtgctggaacacggtaaaaccatgaccgagaaggagatcgtggactatgtggccagccaggttacaaccgcca<br>agaagctgcgcggtggtgttgtgttcgtggacgaggtgcctaaaggactgaccggcaagttggacgcccgcaagatccgcgagattctcatt<br>aaggccaagaagggcggcaagatcgccgtgtaataattctagagtcggggcggccggccgcttcgagcagacatgataagatacattgat<br>gagtttggacaaaccacaactagaatgcagtgaaaaaaatgctttatttgtgaaatttgtgatgctattgctttatttgtaaccattataagctgcaat<br>aaacaagttggatccacgcgccctgtagcggcgcattaagcgcggcgggtgtggtggttacgcgcagcgtgaccgctacacttgccagcgc<br>cctagcgcccgctcctttcgctttcttcccttcctttctcgccacgttcgccggctttccccgtcaagctctaaatcgggggctccctttagggttccga<br>tttagtgctttacggcacctcgaccccaaaaaacttgatttgggtgatggttcacgtagtgggccatcgccctgatagacggtttttcgccctttgac<br>gttggagtccacgttctttaatagtggactcttgttccaaactggtt

Supplementary Table 7. The two detailed PCR amplification procedures (PCR-1 and PCR-2).

PCR-1 procedure

Enzyme step		Temperature (°C)	Time
Initial denaturation		95	3 min
40 cycles	denature	95	30 s
	anneal	68	30 s
	extend	72	1 min
Final extension		72	5 min
Hold		25	∞

PCR-2 procedure

Enzyme step		Temperature (°C)	Time
Initial denaturation		95	3 min
34 cycles	denature	95	30 s
	anneal	58	30 s
	extend	72	1 min
Final extension		72	5 min
Hold		25	∞

Supplementary Table 8. Sequence of primer for PCR amplification.

Oligo Name	Sequence (5'-3')
primer(+)	AGCTCTGCTTATATAGACCTCCCACCGTACACGCCTACCGCCCATTTGCG
primer(-)	ATGGTGAGCAAGGGCGAGGAGCTGTT

Supplementary Table 9. Sequence of single complementary strands (24 nt) of Css EGFP(+).

Oligo Name	Sequence (5'-3')
24nt-1	CCATTTACCGTAAGTTATGTAACG
24nt-2	TCGTTGGGCGGTCAGCCAGGCGGG
24nt-3	ATTATTGACGTCAATGGGCGGGGG
24nt-4	GGCGTTACTATGGGAACATACGTC
24nt-5	TGACGTCAATGGAAAGTCCCTATT
24nt-6	GTTTACCGTAAATACTCCACCCAT
24nt-7	ACTTGATGTACTGCCAAGTGGGCA
24nt-8	GTATCATATGCCAAGTACGCCCCC
24nt-9	CATTTACCGTCATTGACGTCAATA
24nt-10	TACTGGGCATAATGCCAGGCGGGC
24nt-11	GTAGGAAAGTCCCATAAGGTCATG
24nt-12	ACTAATACGTAGATGTACTGCCAA
24nt-13	CGCATCACCATGGTAATAGCGATG
24nt-14	CGCCCATTGATGTACTGCCAAAAC
24nt-15	CCCCGTGAGTCAAACCGCTATCCA
24nt-16	TCAATGGGGTGGAGACTTGGAAAT
24nt-17	TGCCAAAACAAACTCCCATTGACG
24nt-18	TTTGGAAAGTCCCGTTGATTTTGG
24nt-19	AATGGGGCGGAGTTGTTACGACAT
24nt-20	ACACGCCTACCGCCCATTTGCGTC
24nt-21	CTGCTTATATAGACCTCCCACCGT
24nt-22	ATCTGACGGTTCACTAAACCAGCT
24nt-23	ATCTGAGTCCGGTAGCGCTAGCGG
24nt-24	CAGAATTCGAAGCTTGAGCTCGAG
24nt-25	CCGGGCCCGCGGTACCGTCGACTG
24nt-26	CACCATGGTGGCGACCGGTGGATC
24nt-27	GGTGAACAGCTCCTCGCCCTTGCT
24nt-28	CTCGACCAGGATGGGCACCACCCC
24nt-29	GTGGCCGTTTACGTCGCCGTCCAG
24nt-30	GCCCTCGCCGGACACGCTGAACTT
24nt-31	CTTGCCGTAGGTGGCATCGCCCTC
24nt-32	GGTGCAGATGAACTTCAGGGTCAG
24nt-33	CCAGGGCACGGGCAGCTTGCCGGT
24nt-34	GGTCAGGGTGGTCACGAGGGTGGG
24nt-35	GCGGCTGAAGCACTGCACGCCGTA
24nt-36	GTGCTGCTTCATGTGGTCGGGGTA
24nt-37	GGGCATGGCGGACTTGAAGAAGTC
24nt-38	GGTGCGCTCCTGGACGTAGCCTTC
24nt-39	GTTGCCGTCGTCCTTGAAGAAGAT
24nt-40	CTTCACCTCGGCGCGGGTCTTGTA
24nt-41	GTTCACCAGGGTGTCGCCCTCGAA
24nt-42	GTCGATGCCCTTCAGCTCGATGCG
24nt-43	CAGGATGTTGCCGTCCTCCTTGAA
24nt-44	GTAGTTGTACTCCAGCTTGTGCCC
24nt-45	CATGATATAGACGTTGTGGCTGTT
24nt-46	GATGCCGTTCTTCTGCTTGTCGGC
24nt-47	GTGGCGGATCTTGAAGTTCACCTT

24nt-48	CTGCACGCTGCCGTCCTCGATGTT
24nt-49	GTTCTGCTGGTAGTGGTCGGCGAG
24nt-50	CACGGGGCCGTCGCCGATGGGGGT
24nt-51	CAGGTAGTGGTTGTCGGGCAGCAG
24nt-52	TTTGCTCAGGGCGGACTGGGTGCT
24nt-53	GTGATCGCGCTTCTCGTTGGGGTC
24nt-54	GGTCACGAACTCCAGCAGGACCAT
24nt-55	CATGCCGAGAGTGATCCCGGCGGC
24nt-56	GCCGCTTTACTTGTACAGCTCGTC
24nt-57	GGCTGATTATGATCTAGAGTCGCG
24nt-58	GTAAAACCTCTACAAATGTGGTAT
24nt-59	GGTGTGGGAGGTTTTTTAAAGCAA
24nt-60	TTTTATGTTTCAGGTTCAGGGGGA
24nt-61	AGTTAACAACAACAATTGCATTCA
24nt-62	AACCATTATAAGCTGCAATAAACA
24nt-63	TTGTGATGCTATTGCTTTATTTGT
24nt-64	AAAAAAATGCTTTATTTGTGAAAT
24nt-65	TACAGGGCGCGTGGATCCGCAGTG
24nt-66	CACCCGCCGCGCTTAATGCGCCGC
24nt-67	CGGTCACGCTGCGCGTAACCACCA
24nt-68	GCGCTAGGGCGCTGGCAAGTGTAG
24nt-69	AAGGGAAGAAAGCGAAAGGAGCGG
24nt-70	AGCCGGCGAACGTGGCGAGAAAGG
24nt-71	CCCGATTTAGAGCTTGACGGGGAA
24nt-72	TAAATCGGAACCCTAAAGGGAGCC
24nt-73	TGGGGTCGAGGTGCCGTAAAGCAC
24nt-74	AACCATCACCCAAATCAAGTTTTT
24nt-75	ATCAGGGCGATGGCCCACTACGTG
24nt-76	ACGTCAAAGGGCGAAAAACCGTCT
24nt-77	CACTATTAAAGAACGTGGACTCCA
24nt-78	GTTAACCAGTTTGGAACAAGAGTC
24nt-79	TAGCCCGAGATAGGGTTGAGTGTT
24nt-80	AAAATCCCTTATAAATCAAAAGAA
24nt-81	AATGCATGGTACCCCGAAATCGGC
24nt-82	CGTAATTGATTACTATTAATAACT
24nt-83	ATATGGGCTATGAACTAATGACCC
24nt-84	CGGAACTCCATATATGGGCTATGA

Supplementary Table 10. Sequence of one single blocking strands (48 nt) of Css EGFP(+).

Oligo Name	Sequence (5'-3')
48nt-1	CCATTTACCGTAAGTTATGTAACGCAGGATGTTGCCGTCCTCCTTGAA
48nt-2	TCGTTGGGCGGTCAGCCAGGCGGGGTAGTTGTACTCCAGCTTGTGCCC
48nt-3	ATTATTGACGTCAATGGGCGGGGGCATGATATAGACGTTGTGGCTGTT
48nt-4	GGCGTTACTATGGGAACATACGTCGATGCCGTTCTTCTGCTTGTCGGC
48nt-5	TGACGTCAATGGAAAGTCCCTATTGTGGCGGATCTTGAAGTTCACCTT
48nt-6	GTTTACCGTAAATACTCCACCCATCTGCACGCTGCCGTCCTCGATGTT
48nt-7	ACTTGATGTACTGCCAAGTGGGCAGTTCTGCTGGTAGTGGTCGGCGAG
48nt-8	GTATCATATGCCAAGTACGCCCCCCACGGGGCCGTCGCCGATGGGGGT
48nt-9	CATTTACCGTCATTGACGTCAATACAGGTAGTGGTTGTCGGGCAGCAG
48nt-10	TACTGGGCATAATGCCAGGCGGGCTTTGCTCAGGGCGGACTGGGTGCT
48nt-11	GTAGGAAAGTCCCATAAGGTCATGGTGATCGCGCTTCTCGTTGGGGTC
48nt-12	ACTAATACGTAGATGTACTGCCAAGGTCACGAACTCCAGCAGGACCAT
48nt-13	CGCATCACCATGGTAATAGCGATGCATGCCGAGAGTGATCCCGGCGGC
48nt-14	CGCCCATTGATGTACTGCCAAAACGCCGCTTTACTTGTACAGCTCGTC
48nt-15	CCCCGTGAGTCAAACCGCTATCCAGGCTGATTATGATCTAGAGTCGCG
48nt-16	TCAATGGGGTGGAGACTTGGAAATGTAAAACCTCTACAAATGTGGTAT
48nt-17	TGCCAAAACAAACTCCCATTGACGGGTGTGGGAGGTTTTTTAAAGCAA
48nt-18	TTTGGAAAGTCCCGTTGATTTTGGTTTTATGTTTCAGGTTCAGGGGGA
48nt-19	AATGGGGCGGAGTTGTTACGACATAGTTAACAACAACAATTGCATTCA
48nt-20	ACACGCCTACCGCCCATTTGCGTCAACCATTATAAGCTGCAATAAACA
48nt-21	CTGCTTATATAGACCTCCCACCGTTTGTGATGCTATTGCTTTATTTGT
48nt-22	ATCTGACGGTTCACTAAACCAGCTAAAAAAATGCTTTATTTGTGAAAT
48nt-23	ATCTGAGTCCGGTAGCGCTAGCGGTACAGGGCGCGTGGATCCGCAGTG
48nt-24	CAGAATTCGAAGCTTGAGCTCGAGCACCCGCCGCGCTTAATGCGCCGC
48nt-25	CCGGGCCCGCGGTACCGTCGACTGCGGTCACGCTGCGCGTAACCACCA
48nt-26	CACCATGGTGGCGACCGGTGGATCGCGCTAGGGCGCTGGCAAGTGTAG
48nt-27	GGTGAACAGCTCCTCGCCCTTGCTAAGGGAAGAAAGCGAAAGGAGCGG
48nt-28	CTCGACCAGGATGGGCACCACCCCAGCCGGCGAACGTGGCGAGAAAGG
48nt-29	GTGGCCGTTTACGTCGCCGTCCAGCCCGATTTAGAGCTTGACGGGGAA
48nt-30	GCCCTCGCCGGACACGCTGAACTTTAAATCGGAACCCTAAAGGGAGCC
48nt-31	CTTGCCGTAGGTGGCATCGCCCTCTGGGGTCGAGGTGCCGTAAAGCAC
48nt-32	GGTGCAGATGAACTTCAGGGTCAGAACCATCACCCAAATCAAGTTTTT
48nt-33	CCAGGGCACGGGCAGCTTGCCGGTATCAGGGCGATGGCCCACTACGTG
48nt-34	GGTCAGGGTGGTCACGAGGGTGGGACGTCAAAGGGCGAAAAACCGTCT
48nt-35	GCGGCTGAAGCACTGCACGCCGTACACTATTAAAGAACGTGGACTCCA
48nt-36	GTGCTGCTTCATGTGGTCGGGGTAGTTAACCAGTTTGGAACAAGAGTC
48nt-37	GGGCATGGCGGACTTGAAGAAGTCTAGCCCGAGATAGGGTTGAGTGTT
48nt-38	GGTGCGCTCCTGGACGTAGCCTTCAAAATCCCTTATAAATCAAAAGAA
48nt-39	GTTGCCGTCGTCCTTGAAGAAGATAATGCATGGTACCCCGAAATCGGC
48nt-40	CTTCACCTCGGCGCGGGTCTTGTACGTAATTGATTACTATTAATAACT
48nt-41	GTTCACCAGGGTGTCGCCCTCGAAATATGGGCTATGAACTAATGACCC
48nt-42	GTCGATGCCCTTCAGCTCGATGCGCGGAACTCCATATATGGGCTATGA
CB(L1)	TGGGCGGGGGTCGTTGGGCGGTCACCATTTACCGTAAGTTATGTAACG
CB(L2)	TTGGCGTTACTATGGGAACATACGCCATTTACCGTAAGTTATGTAACG
CB(L3)	GGCAGTTTACCGTAAATACTCCACCCATTTACCGTAAGTTATGTAACG
CB(L4)	AGGTCATGTACTGGGCATAATGCCCCATTTACCGTAAGTTATGTAACG
CB(L5)	AACCGCTATCCACGCCCATTGATGCCATTTACCGTAAGTTATGTAACG
CB(L6)	AGCTCTGCTTATATAGACCTCCCACCATTTACCGTAAGTTATGTAACG
CB(M1)	TACGTAGATGTACTGCCAAGTAGGAAGGTCATGTACTGGGCATAATGC
CB(M2)	ATGGTAATAGCGATGACTAATACGATGCCAGGCGGGCCATTTACCGTC
CB(M3)	GATGTACTGCCAAAACCGCATCACTTGACGTCAATAGGGGGCGTACTT
CB(M4)	GGGTGGAGACTTGGAAATCCCCGTCAAGTGGGCAGTTTACCGTAAATA
CB(M5)	GAAAGTCCCGTTGATTTTGGTGCCTCCCTATTGGCGTTACTATGGGAA

CB(R1)	AGCTCTGCTTATATAGACCTCCCACTACCGCCCATTTGCGTCAATGGG
CB(R2)	AGCTCTGCTTATATAGACCTCCCAATTTTGGAAAGTCCCGTTGATTTT
CB(R3)	AGCTCTGCTTATATAGACCTCCCATCAATGGGGTGGAGACTTGGAAAT
CB(R4)	AGCTCTGCTTATATAGACCTCCCAATACGTAGATGTACTGCCAAGTAG
CB(R5)	AGCTCTGCTTATATAGACCTCCCAACTTGGCATATGATACACTTGATG
EB(L1)	CAGCTCCTCGCCCTTGCTCACCATTCGCCGGACACGCTGAACTTGTGG
EB(L2)	CAGCTCCTCGCCCTTGCTCACCATTCGTGCTGCTTCATGTGGTCGGGG
EB(L3)	CAGCTCCTCGCCCTTGCTCACCATGGCGGATCTTGAAGTTCACCTTGA
EB(L4)	CAGCTCCTCGCCCTTGCTCACCATTTACTTGTACAGCTCGTCCATGCC
EB(M1)	TCGGCGCGGGTCTTGTAGTTGCCGTGTTGCCGTCCTCCTTGAAGTCGA
EB(M2)	GACTTGAAGAAGTCGTGCTGCTTCTGCCGTTCTTCTGCTTGTCGGCCA
EB(M3)	TCAGCTTGCCGTAGGTGGCATCGCTGGGTGCTCAGGTAGTGGTTGTCG
EB(M4)	TGCTCACCATGGTGGCGACCGGTGAACCTCTACAAATGTGGTATGGCT
EB(R1)	GCTTCTCGTTGGGGTCTTTGCTCATTACTTGTACAGCTCGTCCATGCC
EB(R2)	AGTTCACCTTGATGCCGTTCTTCTTTACTTGTACAGCTCGTCCATGCC
EB(R3)	ATGTGGTCGGGGTAGCGGCTGAAGTTACTTGTACAGCTCGTCCATGCC
CEB	CCATTTACCGTAAGTTATGTAACGAACCTCTACAAATGTGGTATGGCT
NB(L1)	AAGCAAGTAAAACCTCTACAAATGAATAAACAAGTTAACAACAACAAT
NB(L2)	AAGCAAGTAAAACCTCTACAAATGGCTGGCAAGTGTAGCGGTCACGCT
NB(L3)	AAGCAAGTAAAACCTCTACAAATGGTTGAGTGTTGTTAACCAGTTTGG
NB(L4)	AAGCAAGTAAAACCTCTACAAATGTACCGTAAGTTATGTAACGCGGAA
NB(M1)	GAACGTGGCGAGAAAGGAAGGGAATCGAGGTGCCGTAAAGCACTAAAT
NB(M2)	GCGCGTAACCACCACACCCGCCGCGTCAAAGGGCGAAAAACCGTCTAT
NB(M3)	AACAAGTTAACAACAACAATTGCATAATAACTAATGCATGGTACCCCG
NB(R1)	AACTAATGCATGGTACCCCGAAATTACCGTAAGTTATGTAACGCGGAA
NB(R2)	CAGGGCGATGGCCCACTACGTGAATACCGTAAGTTATGTAACGCGGAA
NB(R3)	TGAAAAAAATGCTTTATTTGTGAATACCGTAAGTTATGTAACGCGGAA
CB(C1)	CCCATAAGGTCATGTACTGGGCATATAGATGTACTGCCAAGTAGGAAAGT
CB(C2)	ATGCCAGGCGGGCCATTTACCGTCACATGGTAATAGCGATGACTAATACG
CB(C3)	TTGACGTCAATAGGGGGCGTACTTGTGATGTACTGCCAAAACCGCATCAC
CB(C4)	GCATATGATACACTTGATGTACTGCGAGTCAAACCGCTATCCACGCCCAT
CB(C5)	CAAGTGGGCAGTTTACCGTAAATACGGGGTGGAGACTTGGAAATCCCCGT
CB(C6)	TCCACCCATTGACGTCAATGGAAAGAAAACAAACTCCCATTGACGTCAAT
CB(C7)	TCCCTATTGGCGTTACTATGGGAACGGAAAGTCCCGTTGATTTTGGTGCC
CB(C8)	ATACGTCATTATTGACGTCAATGGGTGGGGCGGAGTTGTTACGACATTTT
CB(C9)	CGGGGGTCGTTGGGCGGTCAGCCAGCACGCCTACCGCCCATTTGCGTCAA
CB(C10)	GCGGGCCATTTACCGTAAGTTATGTCTGCTTATATAGACCTCCCACCGTA
CB(C11)	AACGCGGAACTCCATATATGGGCTAGATCTGACGGTTCACTAAACCAGCT

Supplementary Table 11. Sequence of block-trigger strands on Css EGFP(+) or Css mCherry(+).

Oligo Name	Sequence (5'-3')
EGFP-block-24 nt	TGCTCACCATGGTGTGGTATGGCT
EGFP-block-28 nt	TGCTCACCATGGTGAATGTGGTATGGCT
EGFP-block-36 nt	TGCTCACCATGGTGGCGATACAAATGTGGTATGGCT
EGFP-block-40 nt	TGCTCACCATGGTGGCGACCTCTACAAATGTGGTATGGCT
EGFP-block-44 nt	TGCTCACCATGGTGGCGACCGGCCTCTACAAATGTGGTATGGCT
EGFP-block-48 nt<br>EB(M4)	TGCTCACCATGGTGGCGACCGGTGAACCTCTACAAATGTGGTATGGCT
EGFP-block-80 nt	TGCTCACCATGGTGGCGACCGGTGGATCCCGGGCCCGCGGTTTTTAAA<br>GCAAGTAAAACCTCTACAAATGTGGTATGGCT
EGFP-block-100 nt	TGCTCACCATGGTGGCGACCGGTGGATCCCGGGCCCGCGGTACCGTCG<br>ACTGTGGGAGGTTTTTAAAGCAAGTAAAACCTCTACAAATGTGGTAT<br>GGCT
EB(M4)(phosphorothioate)	TGCTCACCATGGTGTGGTATGGCT (Phosphorothioate for all bases)

EB(M4)(methylation)	TG(5MdC)T(5MdC)A(5MdC)CATGGTGGCGACCGGTGAACCT(5MdC)TA(5MdC)AAATGTGGTATGG(5MdC)T
Teo-CB(M1)	GTATCATTCATACGTAGATGTACTGCCAAGTAGGAAGGTCATGTACTGGGCATAATGC
CB(M1)T	GCATTATGCCCAGTACATGACCTTCCTACTTGGCAGTACATCTACGTATGAATGATAC
Teo-EB(M4)	AACTCTCAAATGCTCACCATGGTGGCGACCGGTGAACCTCTACAAATGTGGTATGGCT
EB(M4)T	AGCCATACCACATTTGTAGAGGTTCACCGGTCGCCACCATGGTGAGCATTTGAGAGTT
Teo-CB(M2)	TCGATAATCTATGGTAATAGCGATGACTAATACGATGCCAGGCGGGCCATTTACCGTC
CB(M2)T	GACGGTAAATGGCCCGCCTGGCATCGTATTAGTCATCGCTATTACCATAGATTATCGA
Teo-NB(L2)	AGGGAGCTCGAAGCAAGTAAAACCTCTACAAATGGCTGGCAAGTGTAGCGGTCACGCT
NB(L2)T	AGCGTGACCGCTACACTTGCCAGCCATTTGTAGAGGTTTTACTTGCTTCGAGCTCCCT
**Block A:**<br>Teo-CB(M1)	GTATCATTCATACGTAGATGTACTGCCAAGTAGGAAGGTCATGTACTGGGCATAATGC
**Block B:**<br>Teo-CB(M2)	TCGATAATCTATGGTAATAGCGATGACTAATACGATGCCAGGCGGGCCATTTACCGTC
**Block C:**<br>Teo-EB(M4)	AACTCTCAAATGCTCACCATGGTGGCGACCGGTGAACCTCTACAAATGTGGTATGGCT
**Block D:**<br>Teo-48nt-18	GTTGTCAGGCTTTGGAAAGTCCCGTTGATTTTGGTTTTATGTTTCAGGTTCAGGGGGA
**Block E:**<br>Teo-48nt-19	AGCTTTATCTAATGGGGCGGAGTTGTTACGACATAGTTAACAACAACAATTGCATTCA
**Block E1:**<br>Teo-CEB1	ATGTACTCGCCCATTTACCGTAAGTTATGTAACGAACCTCTACAAATGTGGTATGGCT
**Block E2:**<br>Teo-CEB2	TGTCACTATCCCATTTACCGTAAGTTATGTAACGAACCTCTACAAATGTGGTATGGCT
**Input A**	GCATTATGCCCAGTACATGACCTTCCTACTTGGCAGTACATCTACGTATGAATGATAC
**Input B**	GACGGTAAATGGCCCGCCTGGCATCGTATTAGTCATCGCTATTACCATAGATTATCGA
**Input C**	AGCCATACCACATTTGTAGAGGTTCACCGGTCGCCACCATGGTGAGCATTTGAGAGTT
**Input D**	TCCCCCTGAACCTGAAACATAAAACCAAAATCAACGGGACTTTCCAAAGCCTGACAAC
**Input E**	TGAATGCAATTGTTGTTGTTAACTATGTCGTAACAACTCCGCCCCATTAGATAAAGCT
**Input E1**	AGCCATACCACATTTGTAGAGGTTCGTTACATAACTTACGGTAAATGGGCGAGTACAT
**Input E2**	AGCCATACCACATTTGTAGAGGTTCGTTACATAACTTACGGTAAATGGGATAGTGACA

Supplementary Table 12. Sequence of 40 staple strands for the folding of Css EGFP(+).

Oligo Name	Sequence (5'-3')
E-1[9]	TGCGGTTCACCAGGGTGTCGCCCTCTTCACCT
E-1[41]	GTCCTCCTTGAAGTCGATGCCCTTTGGCTGTT
E-2[10]	CGGCGCGGGGTGCGCT
E-0[26]	GTTGCCGTCAGCTCGA
E-2[42]	GTAGTTGTCATGATAT
E-0[58]	TTGTGCCCATGTTGCC
E-3[9]	CCTGGACGCTTCGGGC
E-1[25]	AAGAAGATGTCTTGTA
E-3[41]	AGACGTTGTGTTGTGG
E-1[57]	TTGTCGGCACTCCAGC
E-4[10]	ATGGCGGAGGTAGCGG
E-2[26]	AGTCGTGCCGTCCTTG
E-4[42]	CGGATCTTACGCTGCC
E-2[58]	CCTTGATGTCTTCTGC
E-5[9]	CTGAAGCAACGCCGTA
E-3[25]	GTGGTCGGCTTGAAGA
E-5[41]	GTCCTCGACACGGGGC
E-3[57]	CGAGCTGCGAAGTTCA
E-6[10]	GGTCAGGGTTGCCGGT
E-4[26]	AGGGTGGGTGCTTCAT
E-6[42]	CGTCGCCGGGTTGTCG
E-4[58]	GTTCTGCTGTGGTCGG
E-7[9]	GGTGCAGACTTCAGGG
E-5[25]	CGGGCAGCTGGTCACG
E-7[41]	GGCAGCAGTCGCGCTT
E-5[57]	CAGGTAGTATGGGGGT
E-8[10]	TCAGCTTGCACGCTGA
E-6[26]	GGCATCGCCCAGGGCA
E-8[42]	CTCGTTGGCAGCAGGA
E-6[58]	CTCAGGGCTGGGTGCT
E-10[10]	ACTTGTGGTTACGTCGCCGTCCAGGCTCCTCG
E-7[25]	TCGCCGGACCGTAGGT
E-8[26]	CCATGTGACCTCGCCC
E-7[57]	ACGAACTCGGTCTTTG
E-10[26]	GGATGGGCACCACCCC
E-10[42]	AGCTCGTCGAGTCGCG
E-9[25]	GGTGAACACTCGACCA
E-9[41]	GCCGCTTTACTTGTAC
E-8[58]	ATGATCTACATGCCGAGAGTGATCCGGCGGTC
E-11[57]	TGCTCACCATGGTGGCGACCGGTGAACCTCTACAAATGTGGTATGGCT

Supplementary Table 13. In vitro mixing experiments.

Oligo Name	Sequence (5'-3')
Blocking DNA-Quencher	AATACCCTCACCATTTACCGTAAGTTATGTAACGAGCTCTGCTTA TATAGACCTCCCA-BHQ2
Trigger DNA-Fluorophore	Cy3-TGGGAGGTCTATATAAGCAGAGCTCGTTACATAAC TTACGGTAAATGG TGAGGGTATT
