# Supplementary material for: Circular single-stranded DNA as switchable vector for gene expression in mammalian cells
Source: Nat Commun. 2023 Oct 20;14:6665. doi: 10.1038/s41467-023-42437-6 (PMC10589306; doi:10.1038/s41467-023-42437-6)
Supplement: Supplementary file 3 — Reporting Summary [file 41467_2023_42437_MOESM3_ESM.pdf]

nature portfolio

Corresponding author(s): Jie Song

Last updated by author(s): Oct 8, 2023

Reporting Summary

Nature Portfolio wishes to improve the reproducibility of the work that we publish. This form provides structure for consistency and transparency in reporting. For further information on Nature Portfolio policies, see our Editorial Policies and the Editorial Policy Checklist.

Statistics

For all statistical analyses, confirm that the following items are present in the figure legend, table legend, main text, or Methods section.

n/a	Confirmed	
☐	☒	The exact sample size (*n*) for each experimental group/condition, given as a discrete number and unit of measurement
☐	☒	A statement on whether measurements were taken from distinct samples or whether the same sample was measured repeatedly
☐	☒	The statistical test(s) used AND whether they are one- or two-sided<br>*Only common tests should be described solely by name; describe more complex techniques in the Methods section.*
☒	☐	A description of all covariates tested
☒	☐	A description of any assumptions or corrections, such as tests of normality and adjustment for multiple comparisons
☐	☒	A full description of the statistical parameters including central tendency (e.g. means) or other basic estimates (e.g. regression coefficient) AND variation (e.g. standard deviation) or associated estimates of uncertainty (e.g. confidence intervals)
☐	☒	For null hypothesis testing, the test statistic (e.g. *F*, *t*, *r*) with confidence intervals, effect sizes, degrees of freedom and *P* value noted<br>*Give P values as exact values whenever suitable.*
☒	☐	For Bayesian analysis, information on the choice of priors and Markov chain Monte Carlo settings
☒	☐	For hierarchical and complex designs, identification of the appropriate level for tests and full reporting of outcomes
☒	☐	Estimates of effect sizes (e.g. Cohen's *d*, Pearson's *r*), indicating how they were calculated

Our web collection on statistics for biologists contains articles on many of the points above.

Software and code

Policy information about availability of computer code

Data collection	CytoFLEX LX, Beckman Coulter; Oxford instruments Cypher VRS; OLYMPUS CKX53; Nikon A1 HD25; Amersham ImageQuant 800; PerkinElmer IVIS Lumina III; Nanodrop one (Thermo Fisher)
Data analysis	Graphpad Prism 8.0; FlowJo v10; cadnano v2; Nikon NIS-Elements AR 4.3

For manuscripts utilizing custom algorithms or software that are central to the research but not yet described in published literature, software must be made available to editors and reviewers. We strongly encourage code deposition in a community repository (e.g. GitHub). See the Nature Portfolio guidelines for submitting code & software for further information.

Data

Policy information about availability of data

All manuscripts must include a data availability statement. This statement should provide the following information, where applicable:

- Accession codes, unique identifiers, or web links for publicly available datasets
- A description of any restrictions on data availability
- For clinical datasets or third party data, please ensure that the statement adheres to our policy

The authors declare that the data supporting the findings of this study are available within the article and its Supplementary information file or Source Data file. Source data for each graph has been provided as Source Data 1 (excel), and uncropped gel images have been provided as Source Data 2 (pdf) in the Source Data. Source data are provided with this paper.

Research involving human participants, their data, or biological material

Policy information about studies with human participants or human data. See also policy information about sex, gender (identity/presentation), and sexual orientation and race, ethnicity and racism.

Reporting on sex and gender	not applicable
Reporting on race, ethnicity, or other socially relevant groupings	not applicable
Population characteristics	not applicable
Recruitment	not applicable
Ethics oversight	not applicable

Note that full information on the approval of the study protocol must also be provided in the manuscript.

Field-specific reporting

Please select the one below that is the best fit for your research. If you are not sure, read the appropriate sections before making your selection.

☒ Life sciences ☐ Behavioural & social sciences ☐ Ecological, evolutionary & environmental sciences

For a reference copy of the document with all sections, see nature.com/documents/nr-reporting-summary-flat.pdf

Life sciences study design

All studies must disclose on these points even when the disclosure is negative.

Sample size	Sample size was determined based on standards for experimental cell biology, attempting to have a minimum of N = 3 biological replicates with sufficient reproducibility.
Data exclusions	No data was excluded.
Replication	All experimental findings are reliably reproduced. We have reproduced each experiment at least three times to ensure reliability.
Randomization	For in vivo experiments in mice, animals were randomized to the experimental groups. For in vitro experiments, wells were randomly assigned into each group and all cells were analysed equally.
Blinding	Blinding was not used for cell experiments due to the use of automated collection and analysis systems, where each entire set of cytometry data was acquired and analyzed using identical parameters. Blinding was not performed for fluorescent microscopy experiments as they were not used for direct measurements or statistical analysis to justify blinding, and instead were used as complementary qualitative methods to bulk quantitative methods such as flow cytometry. For animal experiments, investigators were blind to the group allocation during data acquisition and analysis.

Reporting for specific materials, systems and methods

We require information from authors about some types of materials, experimental systems and methods used in many studies. Here, indicate whether each material, system or method listed is relevant to your study. If you are not sure if a list item applies to your research, read the appropriate section before selecting a response.

Materials & experimental systems

n/a	Involved in the study	
☒	☐	Antibodies
☐	☒	Eukaryotic cell lines
☒	☐	Palaeontology and archaeology
☐	☒	Animals and other organisms
☒	☐	Clinical data
☒	☐	Dual use research of concern
☒	☐	Plants

Methods

n/a	Involved in the study	
☒	☐	ChIP-seq
☐	☒	Flow cytometry
☒	☐	MRI-based neuroimaging

Eukaryotic cell lines

Policy information about cell lines and Sex and Gender in Research

Cell line source(s)	C-38 and HCCL-M3 cell lines were kindly provided by Pengfei Zhang (Hangzhou Institute of Medicine, Chinese Academy of Sciences, China). HCT 116 and SW-480 cell lines were kindly provided by Yanlin Song (Xiamen University, China). MDCK cell line was kindly provided by Jinglin Wang (State Key Laboratory of Pathogen and Biosecurity, China). B16 (CL-0029), MCF-7(CL-0149), AC16 (CL-0790), U87-MG (CL-0238), Hela (CL-0101), HELF (L-0281), HEK-293T (CL-0005), Hepa 1-6 (CL-0105), L929 (CL-0137), A549 (CL-0016), 5637 (CL-0002), Ishikawa (CL-0283), LOVO (CL-0144) cell lines were obtained from Procell Life Science & Technology Co., Ltd (Wuhan, China). HIEC-6 (CRL-3266), WRL-68 (CL-48), THLE-3 (CRL-3583), HUVEC (CRL-1730) cell lines were obtained from the American Type Culture Collection (ATCC)
Authentication	The MDCK cell line was authenticated by STR profiling (FuHeng Biology, China).
Mycoplasma contamination	Cell lines were not tested for mycoplasma contamination.
Commonly misidentified lines (See ICLAC register)	No commonly misidentified cell lines were used.

Animals and other research organisms

Policy information about studies involving animals; ARRIVE guidelines recommended for reporting animal research, and Sex and Gender in Research

Laboratory animals	Five BALB/c mice (All 6-week-old females). All mice were housed in temperatures 20 – 25 °C, humidity 30 – 70% and a 12 h light/12 h dark cycle.
Wild animals	The study did not involve wild animals.
Reporting on sex	Sex analysis was not necessary for this study.
Field-collected samples	The study did not involve samples collected from the field.
Ethics oversight	All mice experiments were approved by the Animal Care and Use Committee of Institute of Basic Medicine and Cancer(IBMC), Chinese Academy of Science(2022R0004).

Note that full information on the approval of the study protocol must also be provided in the manuscript.

Plants

Seed stocks	not applicable
Novel plant genotypes	not applicable
Authentication	not applicable

Flow Cytometry

Plots

Confirm that:

- [x] The axis labels state the marker and fluorochrome used (e.g. CD4-FITC).
- [x] The axis scales are clearly visible. Include numbers along axes only for bottom left plot of group (a 'group' is an analysis of identical markers).
- [x] All plots are contour plots with outliers or pseudocolor plots.
- [x] A numerical value for number of cells or percentage (with statistics) is provided.

Methodology

Sample preparation	Cells were digested with trypsin, then eluted with culture medium, and finally washed and resuspended with PBS.

Instrument	CytoFLEX LX Flow Cytometer.
Software	Analysis was carried out using FlowJo 10.
Cell population abundance	10,000 events were analyzed for each sample in each individual experiment.
Gating strategy	Cells were gated first by FSC/SSC, and then single cells were gated on FSC-A/FS-H. Untreated cells were used as a negative control.

☒ Tick this box to confirm that a figure exemplifying the gating strategy is provided in the Supplementary Information.
