# Supplementary material for: Circular single-stranded DNA as switchable vector for gene expression in mammalian cells
Source: Nat Commun. 2023 Oct 20;14:6665. doi: 10.1038/s41467-023-42437-6 (PMC10589306; doi:10.1038/s41467-023-42437-6)

Source data – Fig. 1a

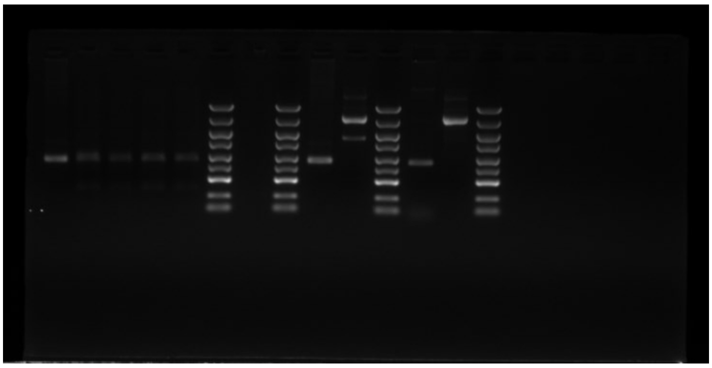

Source data – Fig. 1b

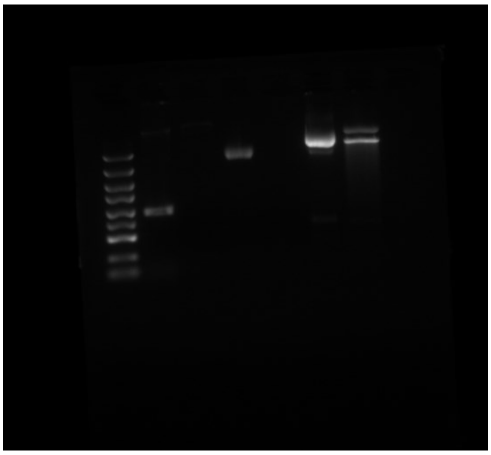

Source data – Fig. 1h

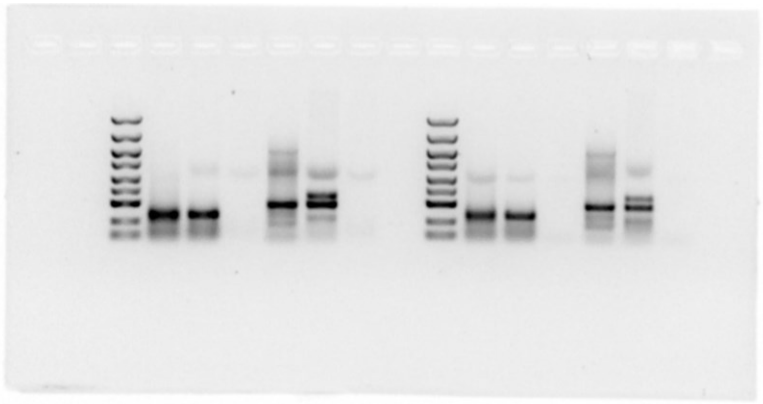

Source data – Supplementary Fig. 2

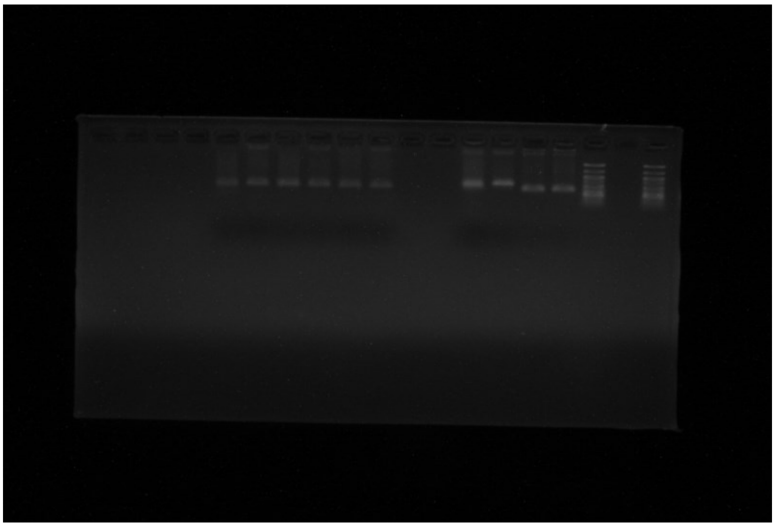

Source data – Supplementary Fig. 3

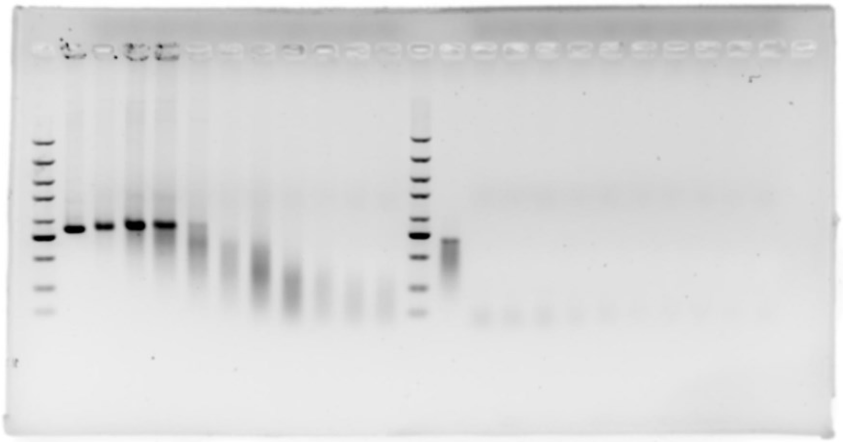

Source data – Supplementary Fig. 10b

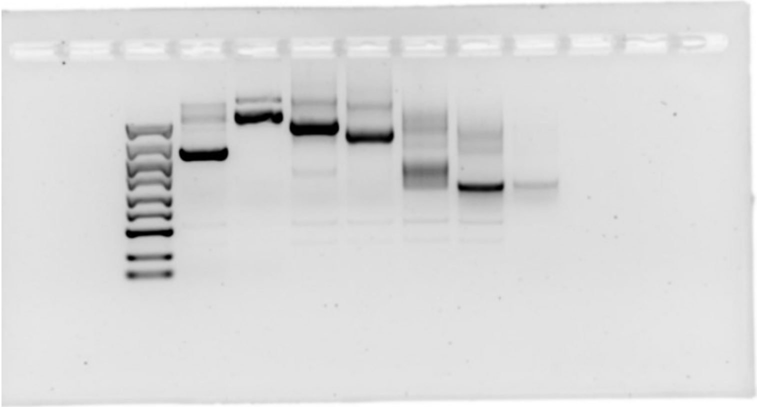

Source data – Supplementary Fig. 29a

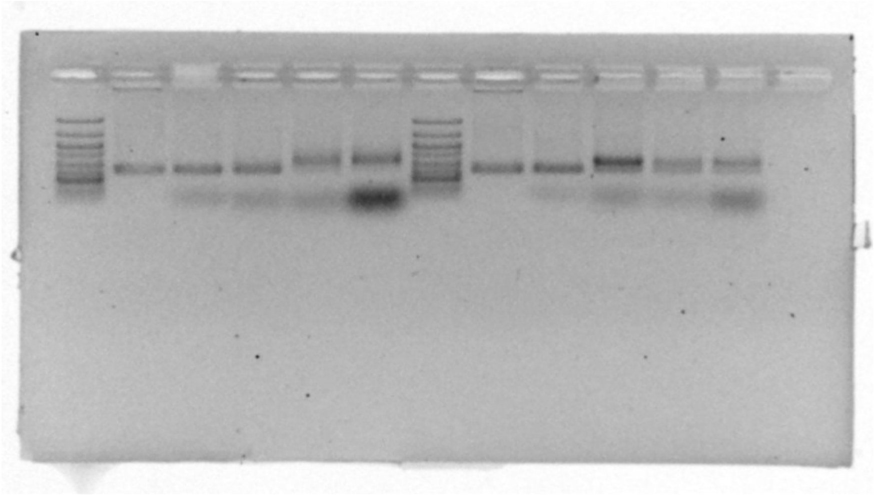

Supplement: Supplementary file 4 — Source Data [file 41467_2023_42437_MOESM4_ESM.zip › Source Data/Source data 2.pdf]
